# Older health and social care workers’ labour market patterns: a 16-year longitudinal study from ages 61–65 to 76–80

**DOI:** 10.1186/s12913-025-13707-4

**Published:** 2025-11-26

**Authors:** Aleksiina Martikainen, Kristina Alexanderson, Pia Svedberg, Kristin Farrants

**Affiliations:** Division of Insurance Medicine, Department of Clinical Neuroscience, Karolinska Institutet, Stockholm, SE-17177 Sweden

**Keywords:** Extended working life, Healthcare workforce, Older workers, Post-retirement work, Sick-leave

## Abstract

**Background:**

Despite the large number of older workers in the health and social care sector, longitudinal, large-scale studies of their labour market situation are lacking. The aim of this study was to examine labour market patterns among older women and men working in the health and social care sector.

**Methods:**

A longitudinal cohort study of the 15,145 women and 6,059 men who, in 2010, were aged 66–70, in paid work at organisations classified as delivering health and social care services and had lived in Sweden throughout 2005–2010. Individually linked register data were used to analyse labour market states across 2005–2020 with sequence- and cluster analyses.

**Results:**

Pronounced sex differences were observed in the occupational distribution at baseline (2010): the largest differences concerned assistant nurses and care assistants (46% of women, 21% of men) and physicians (4% of women, 25% of men). Income data indicated that many worked part-time at some point (5%-51% depending on the year). Women consistently earned less than men, both at baseline (median annual work income 8,199 vs. 10,213 EUR) and throughout the study period. Three distinct clusters of labour market sequences were identified among women and four among men. These clusters differed from each other in timing of work exit, history of sickness absence and disability pension, occupations, and type of care settings. Patterns representing extended working lives were relatively common (51% of all women and 60% of all men) but less common among workers in residential care, as well as among assistant nurses and care assistants, regardless of sex.

**Conclusions:**

The findings highlight the sex segregation of occupations and the income gap in the Swedish labour market and show that extended working lives were less common in certain occupations and care settings. Stakeholders should consider these differences when designing policies aimed at encouraging extended working lives and ensure that women and men have equal opportunities to remain in paid work.

**Supplementary Information:**

The online version contains supplementary material available at 10.1186/s12913-025-13707-4.

## Introduction

### Extending working lives as a response to ageing populations

Many countries are facing a demographic shift towards ageing populations, which has raised concerns about how to maintain a stable workforce and ensure the financial sustainability of welfare systems [1]. To address these challenges, many governments have raised retirement ages and implemented various policy reforms to encourage people to extend their working lives [2–4].

### Who prolongs working life?

Many studies have been conducted on people who retire early, i.e., before reaching the standard retirement age, while people who remain in paid work have received much less attention. It is important to note that there is a degree of selection into continued paid work after standard retirement age, meaning that individuals who exit paid work early are likely to differ from those who remain in several aspects. For example, remaining in paid work after retirement age has been found to be more common among people with higher education [5–7] and without a history of sickness absence and disability pension [6, 7]. In contrast, sickness absence increases the risk of disability pension [8, 9], which is the primary route for exiting paid work before reaching retirement age [10, 11]. Other factors that have been linked to exit from paid work before reaching retirement age include high physical work demands [12, 13], high pension entitlements [14], poor psychosocial working conditions [13], and poor physical and mental health [12, 15, 16]. However, the association between health and retirement appears inconsistent, as highlighted by a recent systematic review [17]. Most of the included studies (*n* = 42) reported better health among those who continued working, some studies (*n* = 21) found non-significant results, while a few (*n* = 6) reported better health among those who did not remain in paid work beyond the standard retirement age [17]. Several factors may mediate this association, including work capacity [18, 19] and opportunities to work (e.g., age discrimination and the attitude of colleagues and supervisors) [18]. Most likely, the decision to remain in or leave paid work is influenced by a variety of factors, including personal preferences, societal norms, legislation, pension eligibility, and broader cultural context. It is also important to note that the age at which someone is considered an “older” worker varies across studies and countries.

### Older workers in Sweden

In Sweden, age 65 has long been regarded as the societally expected retirement age, although there is no official, fixed retirement age [20]. While many still retire at 65, this norm has started to weaken in recent years [21]. Internationally, Sweden has a relatively high proportion of older workers; in 2023, 21% of people over the age 65 remained in paid work, compared to the OECD average of 16% [22]. When looking at specific ages (rather than all people above 65), the rates vary: in 2021, 44% of 67-year-olds, 29% of 70-year-olds, 25% of 72-year-olds, and 19% of 75-year-olds had some income from paid work [23, 24]. Among those who continue working beyond age 65, many work part time and combine work income with pension benefits [25], particularly at ages 66–68 [26]. At higher ages, very few remain in paid work without also drawing pension benefits (e.g., only about 200 individuals aged 75–85 in 2023) [26]. For both women and men who combine work and pension, pension income generally exceeds work income, reflecting that for many people, starting to draw pension happens concurrently with reducing working hours [26]. It should also be noted that in Sweden, drawing a small share of pension can qualify individuals for different pensioner discounts [26], without reducing their working hours, illustrating the complexity of how work income and pension benefits may be combined in practice.

In the public sector, many people shift from permanent contracts before age 65 to hourly contracts after 65 [27]. However, most workers aged 65–74 remain in the same sector in which they worked when aged 55–64 [28]. In general, the Swedish labour market is highly sex-segregated numerically; as in most countries; women and men are concentrated in different occupations and sectors [29, 30]. This pattern continues into older age: among women aged 65–74, the leading sector is health and social care, whereas among men in the same age group, the leading sector is finance, and health and social care sector does not rank among their top five sectors [28]. Within the health and social care sector, the number of workers beyond age 65 varies by occupation, with the highest numbers observed among nurses, physicians, midwives, and biomedical scientists [31].

### Challenges in the health and social care sector

The health and social care sector not only includes a substantial proportion of older workers but is also under growing pressure due to population ageing. As more people live longer with chronic diseases, the demand for health and social care services keeps increasing [32, 33]. Moreover, in Sweden, many current workers in the sector are approaching or have already reached the standard retirement age [32], leading to a need to recruit new employees. Additional challenges include high work demands in the form of time pressure, emotional, mental, or physical demands – that often occur simultaneously [34–36]. These conditions have contributed to high staff turnover, recruitment difficulties, and an increasing number of workers leaving the sector [34–36].

In Sweden, health and social care workers have among the highest sickness absence rates compared to other occupational groups, particularly due to mental diagnoses [37]. The rates are especially high among assistant nurses, personal assistants, psychologists, and cleaning, laundry, and sanitation workers [27]. Women also have higher sickness absence rates than men [27] and are more likely to work part-time in this sector [27, 38, 39], although these patterns are not unique to health and social care but are observed across all sectors in Sweden.

### Why older workers choose to stay in the health and social care sector

Despite the beforementioned challenges, many older health and social care workers remain in the labour force at higher ages, and their motivations appear to be quite similar across qualitative studies conducted in different countries. Common internal motivations for continuing to work include gaining a sense of purpose and meaning [40, 41] and maintaining personal health [40, 42]. External drivers, in turn, can for example involve positive relationships in the work community [41, 42] or financial necessity [40, 43]. Studies investigating factors that would encourage older healthcare workers to extend their working lives have identified increased flexibility over schedule and workload [43–46] and reduced ageism [47] as important motivators. While these qualitative insights increase the understanding of why older health and social care workers can remain in or exit paid work, much less is known about these workers’ actual labour market patterns – who stays, for how long, and what characterises their labour market patterns.

### Current knowledge gaps

A recent systematic literature review shows that most existing research on older health and social care workers originates from Australia, and that there is a lack of studies in the European context [48]. Many prior studies have used cross-sectional designs, making it challenging to distinguish long-term trends from short-term snapshots. In addition, existing research has often focused on one occupational group at a time, such as nurses or physicians, or on samples from specific hospitals or care facilities [48]. As a result, the generalisability of these findings is limited. There is a great need for longitudinal studies that include a broader range of occupations and more representative samples [48]. This type of information is essential, as the timing and pathways of labour market exit among care workers have important implications for healthcare organisations, workforce planning, care delivery, and for developing policies that support sustainable working conditions for older workers in this sector. Such knowledge is especially urgent and relevant in a system already under significant pressure.

### Aim

The aim of this study was to examine labour market patterns among older women and men working in the health and social care sector.

## Method

### Study design and study population

This study was a 16-year longitudinal, population-based cohort study. The focus was specifically on the health and social care workers who had already extended their working lives beyond the standard retirement age in Sweden (65 years). Individuals who exited paid work before or at age 65 were deliberately excluded, as they often represent a distinct group from those who continue working, as described in the Introduction.

All women and men who, in 2010, were aged 66–70, in paid work at organisations classified as delivering health and social care services according to the Swedish Standard Industrial Classification (SNI 2007) [49], and had lived in Sweden throughout 2005–2010 (to reduce bias from recent migration) were included in the study. Applying these inclusion criteria resulted in 15,145 women and 6,059 men (a detailed overview of the criteria is provided in Supplementary Fig. 1). Using 2010 as the baseline, these individuals’ labour market patterns were tracked five years retrospectively (2005–2009) and ten years prospectively (2010–2020), resulting in a 16-year observation period, covering ages 61–65 to 76–80.

### Data sources

Pseudonymized microdata were used, linked at the individual level, from the following nationwide Swedish administrative registers:

- Statistics Sweden’s Longitudinal Integrated Database for Health Insurance and Labour Market Studies (LISA) for information on sociodemographic factors, income, work-related factors, and emigration [50].
- Social Insurance Agency’s Micro Data for the Analysis of Social Insurance (MiDAS) for information on the annual number of sickness absence (SA) (in spells >14 days) and disability pension days [51].
- National Board of Health and Welfare’s Cause of Death register for the year of death [52].

### Swedish context

#### Health and social care sector

Most health and social care services in Sweden are provided within the public sector. Health care is primarily the responsibility of the 21 regions, while social care, including elder care, is managed by the 290 municipalities [53]. However, there are also many private providers that either run their own businesses (e.g., as specialized physicians) or, as in most cases, are contracted by a clinic or a regional or municipal authority to deliver publicly funded healthcare [53]. The health and social care workforce is numerically female dominated and includes a higher proportion of people born outside Sweden compared to other sectors [31]. Most workers are unionized and covered by collective agreements, which govern aspects such as occupational pensions, vacation rights, and working hours.

#### The Swedish social insurance system

All individuals registered as living in Sweden with income from paid work, parental leave allowance, or unemployment benefits are eligible for sickness absence (SA) benefits if disease or injury reduces their work capacity [54]. If the work incapacity is permanent, disability pension (DP) can be granted up to age 65. Both SA and DP can be granted for 25%, 50%, 75%, or 100% of regular working hours [55]. SA covers 80% and DP 64% or lost income, up to a certain level. During this study (2005–2020), SA was limited to 180 days for individuals aged 65–69, unless the Social Insurance Agency expected them to return to work – in which case, additional days could be granted. From age 70, no more than 180 SA days could be granted.

Previously, employees in Sweden had the right to retain their permanent position until age 67 under the Employment Protection Act (LAS), however, this limit was raised to 68 in 2020 [56]. After that age, the employer can choose to extend the employee’s contract. In parallel, Sweden has a flexible pension system. Individuals can claim full or partial pension benefits once they reach the minimum age for each pension component, but it is also possible to delay withdrawals indefinitely, or combine pension benefits with paid work [57]. The minimum age for claiming income-based public pension was 61 until 2019; in 2020 this limit was raised to 62 [58]. Most employees also receive employer-paid occupational pensions, with varying minimum ages depending on the collective agreement [58]. Individuals with minimal or no income are eligible for a so called ‘guarantee pension’, which could be claimed from age 65 during this study period [59]. Residents with low pensions can also apply for income support for the elderly and a housing supplement [60].

### Variables

Distribution of the following variables was examined at baseline in 2010: age, birth country, type of living area, partnership status, educational level, work income, employment sector, type of employment, care setting, occupation, and sickness absence (SA). The categorisation of these variables is shown in Table 1.

### Statistical analyses

All analyses were sex stratified. Descriptive analyses were conducted to examine the distribution of the aforementioned baseline characteristics. The main analyses were conducted with sequence analysis and cluster analysis, which are especially suitable for examining processes over time [61].

#### Definition of the labour market states

Labour market states were defined based on subject-specific knowledge and frequencies in the raw data. For each year, an individual could belong to only one of the following, mutually exclusive states:

A. **Low work income**: Annual work income that was at least (≥) 75% of the income requirement for sickness absence benefits (7,092 − 8,064 SEK depending on the year), but less or equal to (≤) 2 *price base amounts* (78,800 − 89,000 SEK).
B. **Medium/high work income**: Annual work income over (>) 2 *price base amounts* (> 78,800 − 89,000 SEK depending on the year).
C. **Sickness absence and/or disability pension (SA/DP)**: More than 0 SA/DP days and work income of at least (≥) 75% of the income requirement for SA benefits. Individuals who had SA/DP but no work income above this threshold were not considered to be in paid work and were thus included in the state “No work income” (see state E below).
D. **Emigrated or dead**: An individual could belong to this state from 2011 and onward as the inclusion criteria restricted the study population to people who lived in Sweden 2005–2010. If an individual left and re-entered Sweden during the observation period, they were included in this state only for the years when they lived outside Sweden.
E. **No work income**: Annual work income less than (<) 75% of the income requirement for SA benefits.

The *Low work income* and *Medium/high work income* states were defined in relation to each other within the study population. Regardless of labour market state, individuals may have received income from other sources than paid work, such as old-age pension or capital.

#### Sequence analysis

Every individual’s unique sequence of labour market states (A-E) was plotted over the 16-year observation period (2005–2020). Yearly distribution of states and the mean time spent in each state were calculated, transition probabilities were estimated, and the ten most common sequences were plotted separately for all women and men. Entropy was calculated to assess the diversity of labour market states each year. Low entropy indicates that most individuals were in the same state in a given year, while high entropy reflects that individuals were spread out across many different states.

#### Calculation of dissimilarity scores

As preparation for cluster analysis, pairwise dissimilarity scores were calculated between all sequences. There are many dissimilarity measures, and the choice depends on which aspect is prioritized in a given study (e.g., order, timing, or duration of states) [62]. Several dissimilarity measures were tested (Dynamic Hamming, traditional OM with high indel and low substitution cost, and Chi2), but only OM spell produced sufficiently high Average Silhouette Widths (ASW) to distinguish between sequences [63]. The best result was achieved using zero expansion cost, which emphasises the order of states [62].

#### Cluster analysis

Dissimilarity scores were used to group individuals into clusters based on sequence similarity. Among the various clustering methods [61], PAM (Partitioning Around Medoids)/K-medoids was chosen due to its flexibility compared to hierarchical clustering algorithms and its robustness to outliers [64]. Based on several quality measures (see Supplementary Table 1), cluster sizes, and the interpretability of state distribution plots, a three-cluster solution was chosen for women and a four-cluster-solution for men. Among women, the four-cluster solution produced similar ASW (0.65) as the three-cluster solution (0.63), which was ultimately chosen because the fourth cluster did not add meaningful complexity. For each cluster, the yearly distribution of states, entropy, and distribution of baseline characteristics (see Variables) were calculated, and the ten most common sequences plotted.

All analyses were conducted with R Studio version 4.4.2. Sequence analysis and cluster analysis were performed with the package TraMineR [65].

## Results

### Baseline descriptives

Table 1 shows that at baseline (2010), women had a lower median work income (78,200 SEK) than men (97,400 SEK). A smaller proportion of women had a university/college education (44% of women, 56% of men) and were self-employed (5% of women, 10% of men). Occupational distribution varied by sex, with the largest differences observed regarding assistant nurses and care assistants (46% of women, 21% of men), physicians (4% of women, 25% of men), nurses (17% of women, 2% of men), and workers in facility and industrial services (e.g., cleaners and security guards) (4% of women, 14% of men).

**Table 1:** Baseline descriptives in 2010 for all women and men

	Women (*n* = 15,145)	Men (*n* = 6,059)
**Age**		
Mean (SD)	67.3 (1.3)	67.6 (1.4)
Median (Q1-Q3)	67 (66–68)	67 (66–69)
**Birth country**,** n (%)**		
Sweden	13,810 (91.2%)	5,373 (88.7%)
Other Nordic country	726 (4.8%)	227 (3.7%)
EU27 country	388 (2.6%)	245 (4.0%)
Rest of the world	221 (1.5%)	214 (3.5%)
**Type of living area**,** n (%)**		
City	4,810 (31.8%)	1,837 (30.3%)
Town and suburb	6,637 (43.8%)	2,600 (42.9%)
Rural area	3,698 (24.4%)	1,622 (26.8%)
**Partnership status**,** n (%)**		
Married/cohabiting	8,100 (53.5%)	4,524 (74.7%)
Single	7,045 (46.5%)	1,535 (25.3%)
**Educational level**,** n (%)**		
Elementary (including 28 persons with missing information)	2,052 (13.5%)	925 (15.3%)
High school	6,512 (43.0%)	1,731 (28.6%)
University/college	6,581 (43.5%)	3,403 (56.2%)
**Work income**,** including work-related benefits (SEK)**		
Mean (SD)	123,610 (136,700)	197,760 (249,250)
Median (Q1-Q3)	78,200 (31,600 − 171,600)	97,400 (30,600 − 276,300)
**Employment sector**,** n (%)**		
Public	11,542 (76.2%)	4,388 (72.4%)
Private	3,603 (23.8%)	1,671 (27.6%)
**Type of employment**,** n (%)**		
Employee	14,395 (95.0%)	5,476 (90.4%)
Self-employed	750 (5.0%)	583 (9.6%)
**Care setting**,** n (%)**		
Inpatient care	2,962 (19.6%)	1,175 (19.4%)
Outpatient care (including primary care and dental care)	2,564 (16.9%)	1,042 (17.2%)
Other healthcare (e.g., ambulance)	846 (5.6%)	276 (4.6%)
Residential care	4,325 (28.6%)	1,435 (23.7%)
Open social services (e.g., home care, personal assistance)	4,448 (29.4%)	2,131 (35.2%)
**Occupation**,** n (%)**		
Leaders/managers	358 (2.4%)	401 (6.6%)
Physicians	612 (4.0%)	1,505 (24.8%)
Nurses	2,530 (16.7%)	112 (1.8%)
Psychologists and social workers	626 (4.1%)	185 (3.1%)
Assistant nurses and care assistants	6,935 (45.8%)	1,263 (20.8%)
Other health professionals (e.g., physiotherapists and dietitians)	542 (3.6%)	67 (1.1%)
Administration and customer service	1,597 (10.5%)	470 (7.8%)
Education, finance, law, or technology (e.g., lawyers and economists)	1,271 (8.4%)	1,087 (17.9%)
Facility and industrial services (e.g., cleaners and security guards)	550 (3.6%)	850 (14.0%)
Missing information	124 (0.8%)	119 (2.0%)
**Sickness absence (SA)**,** n (%)**		
No	14,674 (96.9%)	5,941 (98.1%)
Yes	471 (3.1%)	118 (1.9%)

Note. SD = Standard Deviation, Q1 = First quartile (25th percentile), Q3 = Third quartile (75th percentile). The study population included all women and men who, in 2010, were aged 66–70, in paid work in the health and social care sector and had lived in Sweden throughout 2005–2010

### Yearly distribution of labour market states

**Fig. 1:**
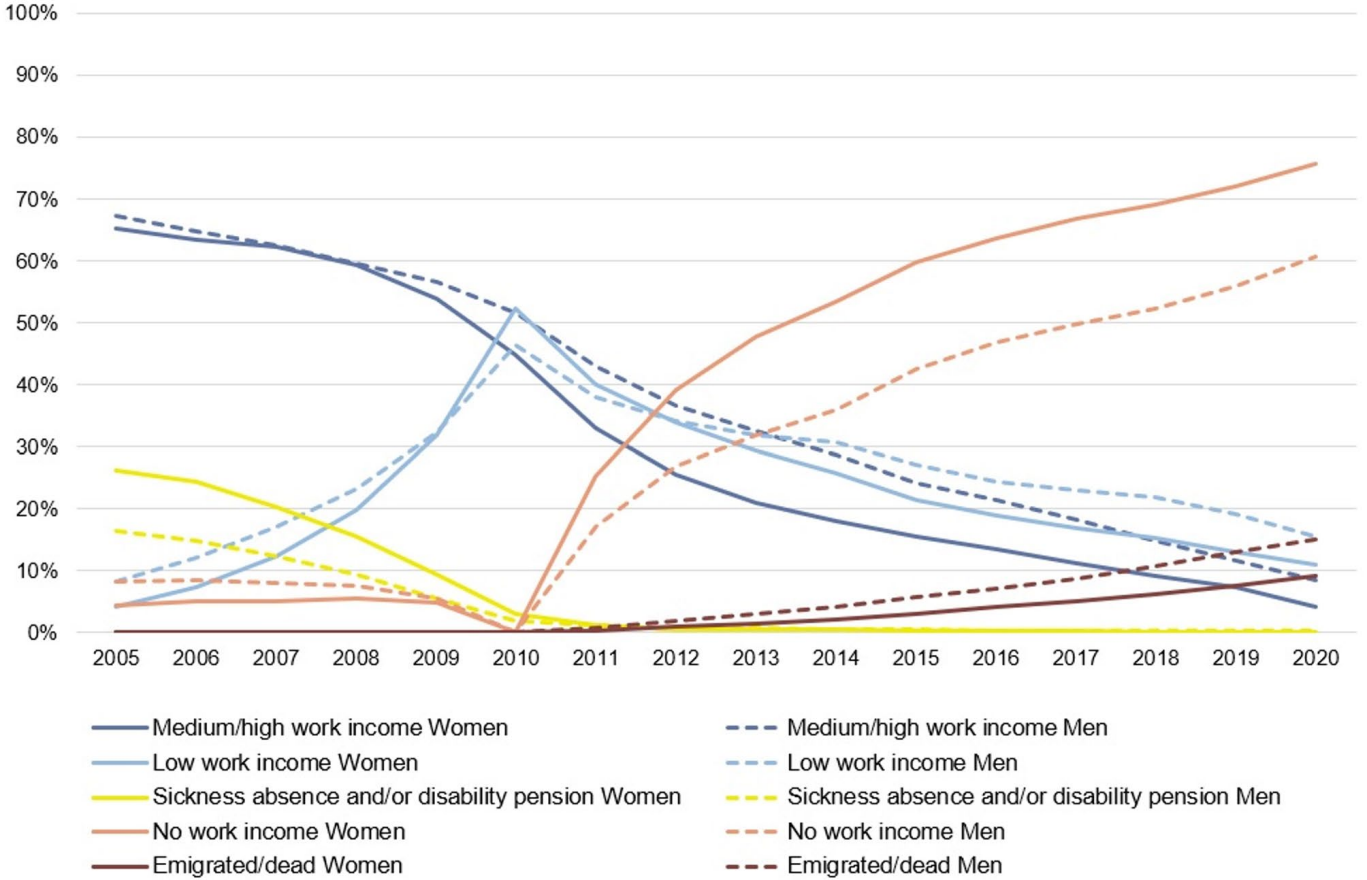
Yearly distribution of the five labour market states among all women (*n* = 15,145) and men (*n* = 6,059). *Note.* The study population included all women and men who, in 2010, were aged 66–70, in paid work in the health and social care sector and had lived in Sweden throughout 2005–2010. Among individuals in the emigrated/dead state, 52 women and 22 men emigrated; the others died. The numbers underlying this figure are presented in Supplementary Table 2

As shown in Fig. 1, the most common state for both women and men during the first five years (2005–2009) was *Medium/high work income*. Over time, the proportion of individuals in this state steadily declined among both sexes, with a clear drop in 2010 (when people were aged 66–70), followed by a continued gradual decrease thereafter. The proportion of individuals on *sickness absence and/or disability pension (SA/DP)* was highest in 2005 (26% of women and 16% of men), gradually declining toward baseline (2010) and dropping sharply thereafter. After baseline, the proportion of individuals with *No work income* increased substantially, especially among women. In the last year (2020), when individuals were aged 76–80, most women (75%) and men (60%) had *No work income*, some had died/emigrated (9% of women and 15% of men), and some were still in paid work (11% of women and 16% of men with *Low work income*; 4% of women and 9% of men with *Medium/high work income*).

### Transitions between states

Supplementary Table 3 shows that the probability of remaining in *No work income* was very high (93% among women, 88% among men), as was the probability of remaining in *Medium/high work income* (76% among women, 81% among men). Among both women and men, the probability of remaining in *SA/DP* was around 50%, while transitioning to *Medium/high work income* was 33%, and to *Low work income* was approximately 14%.

Peaks in entropy (Supplementary Fig. 2) indicate that women were spread across a wider range of labour market states in 2011, and men in 2014–2015. Toward end of the observation period, individuals gradually concentrated into the same states, as reflected by a decline in entropy. This trend was more pronounced among women, while men remained more spread across states, as shown by their relatively stable entropy. The dip in entropy in 2010 is explained by the inclusion criteria, which required all individuals to have work income at baseline.

### Mean time spent in labour market states

On average, men spent one year longer in *Medium/high work income* and half a year longer in *Low work income* than women (Supplementary Fig. 3). In contrast, women spent approximately 1.5 years longer in *No work income* and a few months longer in *SA/DP* than men.

### Labour market sequences

Individual labour market sequences are presented in Supplementary Fig. 4. The ten most common sequences (Supplementary Fig. 5) showed that a lower proportion of women had *Medium/high work income* throughout the entire observation period compared to men (1.3% vs. 3.5%).

### Clusters

**Fig. 2:**
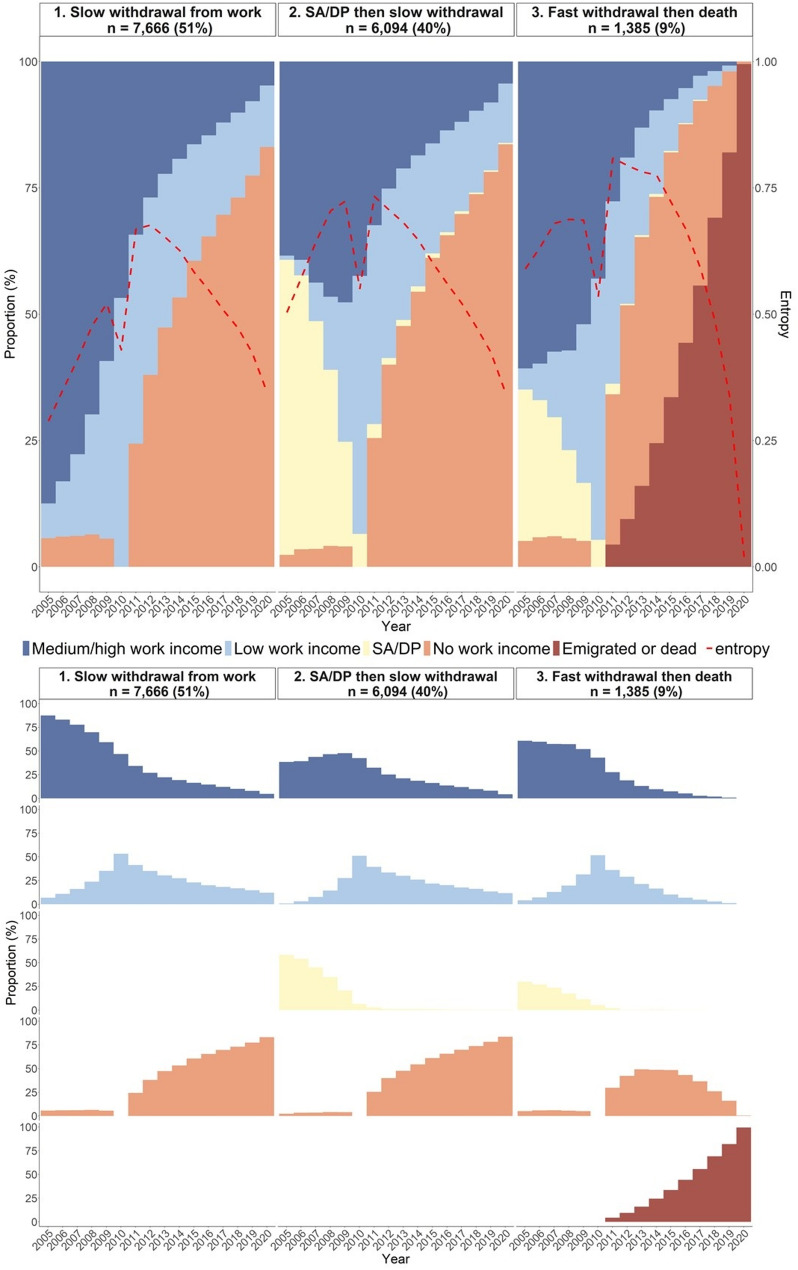
Yearly distribution of labour market states for the three identified clusters among women (*n* = 15,145). *Note.* SA/DP = sickness absence and/or disability pension. The study population included all women and men who, in 2010, were aged 66–70, in paid work in the health and social care sector and had lived in Sweden throughout 2005–2010. The upper part shows the total distribution of states at each year within each cluster. In the lower part, the distribution is decomposed so that each state is shown in its own panel

The identified clusters were named based on the overall patterns of labour market states observed in each cluster. Among women, three clusters were identified (Fig. 2):

1. *Slow withdrawal from work* (51% of women). This cluster was characterised by sustained attachment to paid work. Women gradually transitioned from *Medium/high work income* to *Low work income*, and eventually to *No work income*.
2. *SA/DP then slow withdrawal* (40% of women). This cluster was marked by a relatively high proportion of women initially on *SA/DP*, who were in paid work at baseline (when aged 66–70), and then gradually transitioned to *No work income*.
3. *Fast withdrawal then death* (9% of women). Women in this cluster exited paid work quickly, transitioning from *Medium/high or Low work income* to *No work income* within a few years. After ages 66–70, a growing proportion died or emigrated, reaching nearly 100% by the end of follow-up.

**Fig. 3:**
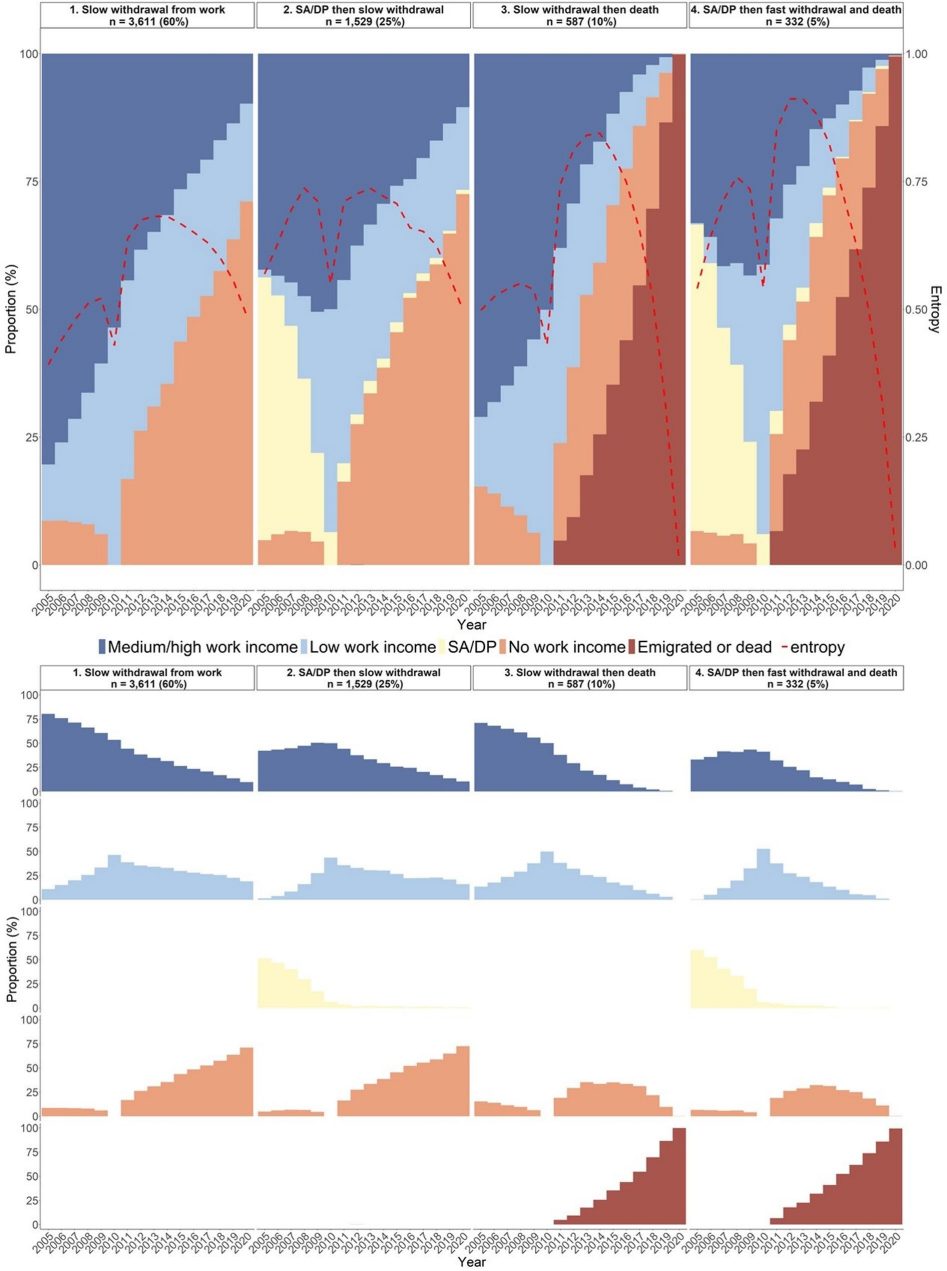
Yearly distribution of labour market states for the four identified clusters among men (*n* = 6,059). *Note.* SA/DP = sickness absence and/or disability pension. The study population included all women and men who, in 2010, were aged 66–70, in paid work in the health and social care sector and had lived in Sweden throughout 2005–2010. The upper part shows the total distribution of states at each year within each cluster. In the lower part, the distribution is decomposed so that each state is shown in its own panel

Among men, four clusters were identified (Fig. 3):

1. *Slow withdrawal from work* (60% of men). This cluster was characterised by sustained attachment to paid work. Men gradually transitioned from *Medium/high work income* to *Low work income*, and eventually to *No work income.*
2. *SA/DP then slow withdrawal* (25% of men). This cluster was marked by a relatively high proportion of men initially on *SA/DP*, which steadily declined toward baseline (ages 66–70), whereafter many men gradually moved to *No work income.*
3. *Slow withdrawal then death* (10% of men). Men in this cluster exited paid work slowly, similar to the first cluster, but were distinguished by a gradually increasing proportion who died or emigrated starting from ages 66–70. By the end of follow-up, nearly all had died or emigrated.
4. *SA/DP then fast withdrawal and death (5% of men).* This cluster was characterised by a relatively high proportion of men initially on *SA/DP*, similar to the second cluster. However, instead of gradually transitioning to *No work income*, men in this cluster exited paid work rapidly and died/emigrated at a fast pace starting around ages 66–70, with nearly 100% having died or emigrated by the end of the follow-up.

Taken together, the largest clusters in both sexes were characterised by a slow withdrawal from paid work, including 51% of women and 60% of men. A higher proportion of women belonged to a cluster marked by a history of SA/DP (40%), although this was also the second largest cluster among men (25%). Smaller clusters in both sexes (9% of women, 5% of men) were characterised by rapid withdrawal from paid work, combined with high mortality at the end of follow-up. The main difference between women and men was the presence of one cluster unique to men (10%), in which a slow withdrawal from work was followed by eventual death without any preceding history of SA/DP.

The ten most common sequences per cluster are presented in Supplementary Fig. 6a (women) and 6b (men). Table 2 shows the baseline characteristics for each cluster in 2010. Many sociodemographic variables, such as age, birth country, and type of living area, were relatively evenly distributed across clusters for both women and men. Educational level, however, varied more notably: among women, the proportion with university/college education ranged from 35% in the “*3. Fast withdrawal then death”* cluster to 45% in the “*1. Slow withdrawal from work”* cluster. Among men, a similar pattern was observed, ranging from 43% in the “*4. SA/DP then fast withdrawal and death”* cluster to 59% in the “*1. Slow withdrawal from work” cluster.*

Differences between clusters were more pronounced regarding care setting and occupation, and these patterns varied by sex. For example, among women, the proportion working in inpatient care was stable across clusters (19–20%), whereas among men, it ranged from 16% in the “*3. Slow withdrawal then death”* and “*4. SA/DP then fast withdrawal and death”* clusters to 21% in the “*1. Slow withdrawal from work*” cluster. Among both women and men, the highest proportion of individuals working in residential care was found in clusters characterised by fast withdrawal from paid work and mortality: 33% in the *“3. Fast withdrawal then death”* cluster among women (compared to 27% and 29% in the other clusters) and 28% in the *“4. SA/DP then fast withdrawal and death”* cluster among men (compared to 23–25% in the other clusters among men). The proportion of men working in education, finance, law, or technology (e.g. lawyers and economists) varied from 11% in the “*4. SA/DP then fast withdrawal and death”* cluster to 20% in the “*1. Slow withdrawal from work”* cluster. Moreover, the proportion of assistant nurses and care assistants was highest in the “*3. Fast withdrawal then death”* cluster among women (53%) and in the “*4. SA/DP then fast withdrawal and death”* cluster among men (30%), and lowest in the “*1. Slow withdrawal from work”* cluster among women (43%) and men (19%).

**Table 2:** Baseline descriptives in 2010 by cluster among women and men

	Women (*n* = 15,145)			Men (*n* = 6,059)			
	1. Slow withdrawal from work (*n* = 7,666)	2. SA/DP then slow withdrawal (*n* = 6,094)	3. Fast withdrawal then death (*n* = 1,385)	1. Slow withdrawal from work (*n* = 3,611)	2. SA/DP then slow withdrawal (*n* = 1,529)	3. Slow withdrawal then death (*n* = 587)	4. SA/DP then fast withdrawal and death (*n* = 332)
**Age**							
Mean (SD)	67.5 (1.4)	67.1 (1.2)	67.4 (1.4)	67.7 (1.4)	67.3 (1.3)	67.9 (1.5)	67.3 (1.3)
Median (Q1-Q3)	67 (66–68)	67 (66–68)	67 (66–69)	67 (66–69)	67 (66–68)	68 (66–69)	67 (66–68)
**Birth country**,** n (%)**							
Sweden	7,028 (91.7%)	5,538 (90.9%)	1,244 (89.8%)	3,236 (89.6%)	1,319 (86.3%)	519 (88.4%)	299 (90.1%)
Other Nordic country	350 (4.6%)	295 (4.8%)	81 (5.8%)	128 (3.5%)	60 (3.9%)	26 (4.4%)	13 (3.9%)
EU27 country	177 (2.3%)	174 (2.9%)	37 (2.7%)	136 (3.8%)	72 (4.7%)	27 (4.6%)	10 (3.0%)
Rest of the world	111 (1.4%)	87 (1.4%)	23 (1.7%)	111 (3.1%)	78 (5.1%)	15 (2.6%)	10 (3.0%)
**Type of living area**,** n (%)**							
City	2,415 (31.5%)	1,944 (31.9%)	451 (32.6%)	1,064 (29.5%)	520 (34.0%)	160 (27.3%)	93 (28.0%)
Town and suburb	3,391 (44.2%)	2,634 (43.2%)	612 (44.2%)	1,574 (43.6%)	627 (41.0%)	259 (44.1%)	140 (42.2%)
Rural area	1,860 (24.3%)	1,516 (24.9%)	322 (23.2%)	973 (26.9%)	382 (25.0%)	168 (28.6%)	99 (29.8%)
**Partnership status**,** n (%)**							
Married/cohabiting	4,372 (57.0%)	3,156 (51.8%)	572 (41.3%)	2,752 (76.2%)	1,137 (74.4%)	401 (68.3%)	234 (70.5%)
Single	3,294 (43.0%)	2,938 (48.2%)	813 (58.7%)	859 (23.8%)	392 (25.6%)	186 (31.7%)	98 (29.5%)
**Educational level**,** n (%)**							
Elementary	1,070 (14.0%)	745 (12.2%)	237 (17.1%)	495 (13.7%)	243 (15.9%)	121 (20.6%)	66 (19.9%)
High school	3,124 (40.8%)	2,730 (44.8%)	658 (47.5%)	990 (27.4%)	428 (28.0%)	190 (32.4%)	123 (37.0%)
University/college	3,472 (45.3%)	2,619 (43.0%)	490 (35.4%)	2,126 (58.9%)	858 (56.1%)	276 (47.0%)	143 (43.1%)
**Annual work income**,** including work-related benefits (SEK)**							
Mean (SD)	125,620 (142,930)	122,140 (129,450)	118,940 (132,350)	201,130 (252,940)	211,460 (256,840)	164,950 (216,430)	155,940 (217,020)
Median (Q1-Q3)	76,200 (30,800 − 175,280)	80,750 (33,300 − 170,000)	79,600 (29,100–162,600)	97,400 (29,650 − 281,450)	114,000 (34,300 − 296,600)	84,900 (29,500 − 231,000)	72,500 (27,050–207,430)
**Employment sector**,** n (%)**							
Public	5,802 (75.7%)	4,662 (76.5%)	1,078 (77.8%)	2,654 (73.5%)	1,095 (71.6%)	410 (69.8%)	229 (69.0%)
Private	1,864 (24.3%)	1,432 (23.5%)	307 (22.2%)	957 (26.5%)	434 (28.4%)	177 (30.2%)	103 (31.0%)
**Type of employment**,** n (%)**							
Employee	7,297 (95.2%)	5,777 (94.8%)	1,321 (95.4%)	3,279 (90.8%)	1,369 (89.5%)	534 (91.0%)	294 (88.6%)
Self-employed	369 (4.8%)	317 (5.2%)	64 (4.6%)	332 (9.2%)	160 (10.5%)	53 (9.0%)	38 (11.4%)
**Care setting**,** n (%)**							
Inpatient care	1,479 (19.3%)	1,213 (19.9%)	270 (19.5%)	740 (20.5%)	291 (19.0%)	92 (15.7%)	52 (15.7%)
Outpatient care (including primary care and dental care)	1,359 (17.7%)	1,033 (17.0%)	172 (12.4%)	603 (16.7%)	300 (19.6%)	86 (14.7%)	53 (16.0%)
Other healthcare (e.g., ambulance)	426 (5.6%)	347 (5.7%)	73 (5.3%)	160 (4.4%)	75 (4.9%)	25 (4.3%)	16 (4.8%)
Residential care	2,079 (27.1%)	1,789 (29.4%)	457 (33.0%)	828 (22.9%)	370 (24.2%)	145 (24.7%)	92 (27.7%)
Open social services (e.g., home care, personal assistance)	2,323 (30.3%)	1,712 (28.1%)	413 (29.8%)	1,280 (35.4%)	493 (32.2%)	239 (40.7%)	119 (35.8%)
**Occupation**,** n (%)**							
Leaders/managers	214 (2.8%)	113 (1.9%)	31 (2.2%)	277 (7.7%)	71 (4.6%)	42 (7.2%)	11 (3.3%)
Physicians	316 (4.1%)	249 (4.1%)	47 (3.4%)	914 (25.3%)	415 (27.1%)	111 (18.9%)	65 (19.6%)
Nurses	1,371 (17.9%)	983 (16.1%)	176 (12.7%)	53 (1.5%)	41 (2.7%)	< 3%*	< 3%*
Psychologists and social workers	319 (4.2%)	272 (4.5%)	35 (2.5%)	111 (3.1%)	50 (3.3%)	< 3%*	< 3%*
Assistant nurses and care assistants	3,286 (42.9%)	2,912 (47.8%)	737 (53.2%)	685 (19.0%)	337 (22.0%)	141 (24.0%)	100 (30.1%)
Other health professionals (e.g., physiotherapists and dietitians)	250 (3.3%)	246 (4.0%)	46 (3.3%)	37 (1.0%)	< 3*	< 3%*	< 3%*
Administration and customer service	843 (11.0%)	609 (10.0%)	145 (10.5%)	287 (7.9%)	104 (6.8%)	47 (8.0%)	32 (9.6%)
Education, finance, law, or technology (e.g., lawyers and economists)	717 (9.4%)	457 (7.5%)	97 (7.0%)	737 (20.4%)	207 (13.5%)	108 (18.4%)	35 (10.5%)
Facility and industrial services (e.g., cleaners and security guards)	285 (3.7%)	218 (3.6%)	47 (3.4%)	454 (12.6%)	250 (16.4%)	87 (14.8%)	59 (17.8%)
Missing information	65 (0.8%)	35 (0.6%)	24 (1.7%)	56 (1.6%)	29 (1.9%)	23 (3.9%)	11 (3.3%)
**Sickness absence (SA)**,** n (%)**							
No	7,666 (100%)	5,697 (93.5%)	1,311 (94.7%)	3,611 (100%)	1,431 (93.6%)	587 (100.0%)	312 (94.0%)
Yes	0 (0%)	397 (6.5%)	74 (5.3%)	0 (0%)	98 (6.4%)	0 (0.0%)	20 (6.0%)

Note. SD = Standard Deviation, Q1 = First quartile (25th percentile), Q3 = Third quartile (75th percentile), < 3%* = too few to show. The study population included all women and men who, in 2010, were aged 66–70, in paid work in the health and social care sector and had lived in Sweden throughout 2005–2010

## Discussion

This was a 16-year longitudinal, population-based cohort study including all 15,145 women and 6,059 men who worked in health and social care organisations at ages 66–70 (in 2010), and who had lived in Sweden throughout 2005–2010. To the best of our knowledge, this is the first study to map labour market patterns among older health and social care workers on this scale and with such detailed, longitudinal data. As a result, comparisons to prior literature are rather limited and complicated by the varying age ranges used to define “older” workers.

### Summary of the results

The results revealed that the income gap between women and men [66] and the numerical sex segregation of occupations [29, 30] that are typical for working-age population persisted even at higher ages in Sweden. Further, three clusters of distinct labour market patterns were identified among women and four among men; the most common cluster was extended working lives (51% of women, 60% of men), followed by a cluster with a history of SA/DP but who still extended their working lives to some degree (40% of women, 25% of men), and a minority who exited paid work quickly and died/emigrated at the end of follow-up (9% of women, 5% of men). In addition, a unique cluster was identified among men, in which they extended their working lives and died/emigrated at the end of follow-up without a history of SA/DP (10% of men).

Further analyses showed that the clusters characterised by the extended working life (the most common cluster for women and men) included a higher proportion of female nurses, male physicians, women working in outpatient care (e.g., primary care), men working in inpatient care, and individuals (both women and men) working with education, finance, law, and technology. In contrast, clusters characterised by fast withdrawal from paid work, followed by death/emigration at the end of follow-up, included a higher proportion of assistant nurses/care assistants and residential care workers, both among women and men. Altogether, the results suggest that work-related characteristics among older health and social care workers may influence both the timing of work exit and later health outcomes such as mortality. Below, we discuss labour market patterns both at the beginning and end of the observation period, investigate the unique group among men in more detail, and discuss possible explanations for the differences between the identified clusters.

### Prior sickness absence and/or disability pension

While most women (65%) and men (67%) had *Medium/high work income* at the start of the observation period in 2005 (i.e., 5 years before inclusion), having sickness absence/disability pension (SA/DP) was also relatively common (26% of women and 16% of men in 2005). Individuals initially on SA/DP had a 33% probability of transitioning to *Medium/high work income*, and a 14% probability of transitioning to *Low work income*, among both women and men. The reasons for these returns remain unclear and could reflect methodological aspects and/or real-life circumstances. Methodologically, these transition rates were likely influenced by the inclusion criteria, which required individuals to be in paid work at baseline in 2010, and the low threshold for inclusion in the SA/DP state (> 0 days per year). However, other explanations are also possible, such as successful rehabilitation during SA/DP that enabled return to work, or alternatively, job changes that were a better fit for individuals initially on SA/DP. Nevertheless, it is possible that people with very high SA/DP rates exited paid work before the baseline and were thus not included in this study.

### Gradual withdrawal from paid work

The results showed that most women and men had *Medium/high work income* from ages 61–65 to 65–69 (from 2005 to 2009). A substantial proportion of individuals remained in paid work for at least some years after ages 66–70, which aligns with prior studies [6, 7]. Transitioning to *Low work income* was relatively common, likely reflecting a transition to part-time work, as even relatively low-salaried full-time workers would typically qualify for the *Medium/high work income* state. This suggests that many health and social care workers in Sweden gradually exit from paid work, rather than experience retirement as a clear cut, one-time event. Whether this indicates a preference to continue working, financial necessity, or reduced capacity to work full-time, for example due to unsustainable working conditions, cannot be answered by this study. However, this could be explored in future research, for example by using data from questionnaires or interviews, in addition to register data. Another factor that can influence continued participation in paid work at older ages is ageism [67]. Most likely, a combination of individual, organisational, and societal factors act as push or pull factors influencing the decision to leave or stay in paid work.

### Unique cluster among men

Notably, one cluster was identified only among men (“*3. Slow withdrawal then death”*). Men in this cluster (10%) had a relatively direct transition from *Medium/high* or *Low income* to *No work income*, followed by death/emigration, and had no preceding SA/DP. Baseline characteristics revealed (Table 2) that this cluster was distinguished by a higher proportion of men working in open social services (e.g., home care, personal assistance) and with elementary education. The latter was also seen in cluster “*4. SA/DP then fast withdrawal and death”*, in which men died/emigrated at the end of follow-up. Possible explanations for this result could include cumulative exposure to physically demanding work in open social services or broader socioeconomic disadvantages associated with lower education. However, these findings also raise questions about potential unmeasured factors, such as undetected health issues, differences in health-seeking behaviour, or lifestyle.

### Occupational and care setting differences

The results indicated that the distribution of occupations and care settings varied considerably across clusters. Due to the observational nature of the cluster analysis, this study cannot answer why this was the case. However, potential explanations could include differences in job characteristics, such as work demands, job control, and flexibility across care settings and occupations. For example, a recent Swedish study found that physicians reported the highest quantitative demands, registered nurses the highest emotional demands, while nursing assistants faced the greatest effort-reward imbalance, work-private life interference, and the lowest levels of job control [68]. The same study also showed that experiences of work demands within these occupations varied by years of experience and sector (private vs. public). Such differences in demands and resources may be linked to who is able and willing to prolong their working lives. This interpretation would align with previous studies showing that high physical work demands [69–71] and low job control [72] are associated with earlier labour market exit, whereas people in more advantaged positions have better opportunities to prolong their working lives [73]. Over time, occupation-specific differences may accumulate, together with broader life-course advantages and disadvantages in labour market attachment and career stability [74]. In combination, these factors can contribute to later-life inequalities and decisions about extending working life. Addressing these inequalities is crucial at the policy level, especially as the health and social care sector is already under pressure due to an ageing population and the resulting increase in care needs of the public, as well as ongoing challenges in recruiting and retaining staff.

### Methodological reflections

The only dissimilarity measure that provided sufficiently high validity scores to distinguish between clusters was OMspell. This suggests that individuals often transitioned between labour market states at similar time points and remained in each state for comparable durations, but that the order in which they experienced the states varied. This pattern is likely influenced by the fact that some individuals did not experience certain states at all (e.g., SA/DP and death/emigration). The lack of detectable dissimilarities in timing and duration could also reflect the influence of strong age norms and legislation in Sweden (such as the Employment Protection Act, LAS), regulating for example how long people can retain permanent positions.

### Strengths and limitations

This study has several strengths. The use of high-quality [75, 76], individually linked data from three nationwide registers enabled a 16-year observation period and the inclusion of all occupational groups within the Swedish health and social care sector. The large, population-based cohort allowed in-depth analyses of several variables. As a result, this study is the first of its kind, generating new knowledge about a growing group of older workers in this sector. Another strength is that only individuals who were in paid work at baseline were included in this study, which ensured a comparable starting point for studying labour market patterns. Importantly, there was no loss to follow-up or drop-outs, and the data coverage was nearly complete, with only some missing information on occupations.

However, some limitations can be noted. Because the available register data included only annual work income (and not, for example, number of days or hours worked, or full-or part-time status), it was not possible to distinguish between full-time and part-time work. As a result, the definition of income states (Low work income and Medium/high work income) required setting cut-off limits based solely on annual income, and alternative thresholds could have produced different categorizations. The lowest limit to be classified as being in paid work was deliberately set low to capture even those workers who had low or intermittent work income, for example from weekend shifts or holiday cover. This approach was particularly important given that many workers in this sector had relatively low work incomes. A higher income limit would have excluded a large proportion of older workers who were still engaged in paid work to some extent. Individuals in the *Low work income* state most likely worked part-time, as their annual income was too low to be achieved through full-time work in Sweden. In contrast, individuals in the *Medium/high work income* state represent a heterogeneous group with diverse income levels and could have worked either full- or part-time. Nevertheless, all individuals in the *Low* and *Medium/high work income* states were engaged in paid work to some degree, which was the main focus of this study.

Further, choosing the number of clusters is fundamentally a subjective decision in any cluster analysis, making transparency around these choices critical. Finally, the LISA register has some lag in updating occupational codes; large companies report updates annually, while small companies report less frequently (approximately every four years). This may have led to some misclassification of occupations, although occupation was not directly used in the sequence or cluster analyses.

## Conclusions

Extended working lives were relatively common among health and social care workers aged 66–71, involving about half of women (51%) and a majority of men (60%). However, opportunities to remain in paid work varied by occupation and care setting. Extended working life was more common among women working in outpatient care, men working in inpatient care, male physicians, female nurses, and individuals (both women and men) working with education, finance, law, or technology (e.g., lawyers and economists). In contrast, extended working lives were less common among residential care workers and assistant nurses/care assistants, regardless of sex. These differences point to inequalities and possible accumulation of diverse work exposures throughout the life course, which can ultimately result in unequal possibilities to remain in paid work at higher ages. Policies that address differences in working conditions are needed to ensure that health and social care workers have equal opportunities to extend their working lives in this sector. Ensuring equal opportunities will also require confronting underlying sex differences, for instance in income. Such measures are especially critical in the near future, given population ageing and the growing care needs of the public.

## Supplementary Information

Below is the link to the electronic supplementary material.

Supplementary Material 1

## Data Availability

The data used in this study is administered by the Division of Insurance Medicine, Karolinska Institutet, and cannot be made public. According to the General Data Protection Regulation, the Swedish law SFS 2018:218, the Swedish Data Protection Act, the Swedish Ethical Review Act, and the Public Access to Information and Secrecy Act, these types of sensitive and confidential data can only be made available, after legal review, for researchers who meet the criteria for access. Readers may contact Professor Ellenor Mittendorfer-Rutz (ellenor.mittendorfer-rutz@ki.se) regarding the data.

## Abbreviation

SA/DP: Sickness absence and/or disability pension

## References

[1] OECD. Working better with age, ageing and employment policies. Paris: OECD Publishing. 2019. Available from: 10.1787/c4d4f66a-en

[2] Soosaar O, Puur A, Leppik L. Does Raising the pension age prolong working life? Evidence from pension age reform in Estonia. J Pension Econ Finance. 2021;20(2):317–35. 10.1017/s1474747220000244.

[3] Kuitto K, Helmdag J. Extending working lives: how policies shape retirement and labour market participation of older workers. Social Policy Adm. 2021;55(3):423–39. 10.1111/spol.12717.

[4] Focacci CN, Oylu G, Motel-Klingebiel A, Kelfve S. The value of pension reforms for late working life: evidence from Sweden. Int J Sociol Soc Policy. 2023;43(13/14):79–89. 10.1108/ijssp-02-2023-0038.

[5] Leinonen T, Chandola T, Laaksonen M, Martikainen P. Socio-economic differences in retirement timing and participation in post-retirement employment in a context of a flexible pension age. Aging Soc. 2020;40(2):348–68. 10.1017/S0144686X18000958.

[6] Farrants K, Head J, Alexanderson K. Trends in associations between sickness absence before the age of 65 and being in paid work after the age of 65: prospective study of three total population cohorts. J Aging Soc Policy. 2022;35(2):197–220. 10.1080/08959420.2021.2022950. 35114914 10.1080/08959420.2021.2022950

[7] Martikainen A, Svensson Alavi A, Alexanderson K, Farrants K. Associations of sickness absence and disability pension due to mental and somatic diagnoses when aged 60–64 with paid work after the standard retirement age; a prospective population-based cohort study in Sweden. BMC Public Health. 2021;21(1):2322. 10.1186/s12889-021-12382-4. 34969394 10.1186/s12889-021-12382-4 PMC8717651

[8] Klein J, Reini K, Saarela J. Sickness absence and disability pension in the very long term: A Finnish Register-Based study with 20 years Follow-Up. 2021;9. 10.3389/fpubh.2021.556648 10.3389/fpubh.2021.556648 PMC7956975 33732671

[9] Kivimäki M, Ferrie JE, Hagberg J, Head J, Westerlund H, Vahtera J, et al. Diagnosis-specific sick leave as a risk marker for disability pension in a Swedish population. J Epidemiol Commun Health. 2007;61(10):915–20. 10.1136/jech.2006.055426. 10.1136/jech.2006.055426 PMC2652975 17873230

[10] van Rijn RM, Robroek SJ, Brouwer S, Burdorf A. Influence of poor health on exit from paid employment: a systematic review. Occup Environ Med. 2014;71(4):295–301. 10.1136/oemed-2013-101591. 24169931 10.1136/oemed-2013-101591

[11] Reeuwijk KG, van Klaveren D, van Rijn RM, Burdorf A, Robroek SJ. The influence of poor health on competing exit routes from paid employment among older workers in 11 European countries. Scand J Work Environ Health. 2017;43(1):24–33. 10.5271/sjweh.3601. 27829251 10.5271/sjweh.3601

[12] Sewdas R, Thorsen SV, Boot CRL, Bjørner JB, Van der Beek AJ. Determinants of voluntary early retirement for older workers with and without chronic diseases: A Danish prospective study. Scand J Public Health. 2020;48(2):190–9. 10.1177/1403494819852787. 31319774 10.1177/1403494819852787 PMC7042495

[13] Sundstrup E, Thorsen SV, Rugulies R, Larsen M, Thomassen K, Andersen LL. Importance of the working environment for early retirement: prospective cohort study with register Follow-Up. Int J Environ Res Public Health. 2021;18(18):1–13. 10.3390/ijerph18189817. 10.3390/ijerph18189817 PMC8472036 34574740

[14] Kuhn U, Grabka MM, Suter C. Early retirement as a privilege for the rich? A comparative analysis of Germany and Switzerland. Adv Life Course Res. 2021;47. 10.1016/j.alcr.2020.100392. 10.1016/j.alcr.2020.100392 36695149

[15] Homaie Rad E, Rashidian A, Arab M, Souri A. Comparison the effects of poor health and low income on early retirement: a systematic review and meta-analysis. Ind Health. 2017;55(4):306–13. 10.2486/indhealth.2017-0010. 28484145 10.2486/indhealth.2017-0010 PMC5546840

[16] Topa G, Depolo M, Alcover CM. Early retirement: A Meta-Analysis of its antecedent and subsequent correlates. Front Psychol. 2017;8:2157. 10.3389/fpsyg.2017.02157. 29354075 10.3389/fpsyg.2017.02157 PMC5759094

[17] Farrants K, Dervish J, Marklund S, Alexanderson K. Health and morbidity among people in paid work after 64 years of age: A systematic review. Social Sci Humanit Open. 2023;8(1):100571. 10.1016/j.ssaho.2023.100571.

[18] de Wind A, Geuskens GA, Ybema JF, Bongers PM, van der Beek AJ. The role of ability, motivation, and opportunity to work in the transition from work to early retirement – testing and optimizing the early retirement model. Scand J Work Environ Health. 2015;124–35. 10.5271/sjweh.3468. 10.5271/sjweh.3468 25393088

[19] Hasselhorn HM, Leinonen T, Bültmann U, Mehlum IS, du Prel JB, Kiran S, et al. The differentiated roles of health in the transition from work to retirement - conceptual and methodological challenges and avenues for future research. Scand J Work Environ Health. 2022;48(4):312–21. 10.5271/sjweh.4017. 35239972 10.5271/sjweh.4017 PMC9524164

[20] The Swedish Social Insurance Agency. Förlängt arbetsliv - förutsättningar, utmaningar och konsekvenser: Rapport från forskarseminariet i Umeå 15–16 januari 2020. [Extended Working Life - Prerequisites, Challenges, and Consequences: Report from the Research Seminar in Umeå, January 15–16, 2020] (In Swedish). 2020. Available from: https://www.forsakringskassan.se/download/18.7fc616c01814e179a9f32d/1656661298206/forlangt-arbetsliv-forutsattningar-utmaningar-och-konsekvenser-socialforsakringsrapport-2020-5.pdf

[21] Fondberg R, Wikmark Kreuger L. Pensionsåldrar och arbetslivets längd - svar på regleringsbrevsuppdrag 2023 [Retirement Ages and the Length of Working Life - Response to the Regulatory Letter Assignment 2023] (In Swedish). 2023. Available from: https://www.pensionsmyndigheten.se/statistik/publikationer/pensionsaldrar-arbetslivets-langd-2022/pensionsaldrar-arbetslivets-langd-2022.pdf

[22] OECD. Labour force participation rate [Internet]. n.d. Available from: https://www.oecd.org/en/data/indicators/labour-force-participation-rate.html?oecdcontrol-f42fb73652-var3=2023%26oecdcontrol-48dba69563-var6=Y_GE65%26oecdcontrol-6c004461ab-var1=OECD%257CAUS%257CAUT%257CBEL%257CCAN%257CCHL%257CCOL%257CCRI%257CCZE%257CDNK%257CEST%257CFIN%257CFRA%257CDEU%257CGRC%257CHUN%257CISL%257CIRL%257CISR%257CITA%257CJPN%257CKOR%257CLVA%257CLTU%257CLUX%257CMEX%257CNLD%257CNZL%257CNOR%257CPOL%257CPRT%257CSVK%257CSVN%257CESP%257CSWE%257CCHE%257CTUR%257CGBR%257CUSA%257COECD_REP

[23] Alert Senior. Allt fler vill jobba extra efter pension [More and more people want to work after retirement] n.d. [cited 2025 30 Jun]. Available from: https://www.alertsenior.se/jobba-som-pensionar/

[24] Alecta. Jobbonärer i siffror [Statistics on jobbonaires, i.e., people who combine work and pension benefits] n.d. [cited 2025 10 June]. Available from: https://www.alecta.se/jobbonarer/jobbonarer-i-siffror

[25] Statistics Sweden [SCB]. Var tredje 67-åring arbetar [Every third 67-year-old works] (In Swedish). 2017 [cited 2025 27 March]. Available from: https://www.scb.se/hitta-statistik/artiklar/2017/Var-tredje-67-aring-arbetar/

[26] Wiese L. Jobbonärer 2025: En analys av trender och effekter [Working retirees 2025: An analysis of trends and effects]. 2025. Available from: https://www.pensionsmyndigheten.se/statistik/publikationer/Jobbonarer-2025-En-analys-av-trender-och-effekter/

[27] Sveriges Kommuner och Regioner [Sweden’s municipalities and regions]. Personalen i välfärden: Personalstatistik för kommuner och regioner 2021 [The Welfare Staff: Personnel Statistics for Municipalities and Regions 2021]. Stockholm: 2022. Available from: https://extra.skr.se/skr/tjanster/rapporterochskrifter/publikationer/personalenivalfarden2021.65353.html

[28] Statistics Sweden. Äldres deltagande på arbetsmarknaden, 2001–2023 [Older people’s participation in the labor market, 2001–2023]: 2024. Available from: https://www.scb.se/contentassets/74e8d3d1dcae484093292268caf9ed94/am0401_2024a01_amftbr2401.pdf

[29] Gonäs L, Wikman A, Vaez M, Alexanderson K, Gustafsson K. Changes in the gender segregation of occupations in Sweden between 2003 and 2011. Scand J Publ Health. 2019;47(3):344–7. 10.1177/1403494819831910. 10.1177/1403494819831910 30977438

[30] Gonäs L, Wikman A, Vaez M, Alexanderson KA-O, Gustafsson K. Gender segregation of occupations and sustainable employment: A prospective population-based cohort study. Scand J Public Health. 2019;47(3):348–56. 10.1177/1403494818785255. 29974817 10.1177/1403494818785255

[31] Sveriges Kommuner och Regioner [Sweden’s municipalities and regions]. Personalen i välfärden: Personalstatistik för kommuner och regioner 2023 [Staff in the Welfare Sector: Personnel Statistics for Municipalities and Regions 2023]. Stockholm: 2024. Available from: https://extra.skr.se/skr/tjanster/rapporterochskrifter/publikationer/personalenivalfarden2023.80024.html

[32] Sveriges Kommuner och Regioner [Sweden’s municipalities and regions]. Välfärdens kompetensförsörjning: Personalprognos 2023–2033 och strategier för att klara kompetensförsörjningen [Welfare Competence Supply: Staff Forecast 2023–2033 and Strategies to Manage Competence Supply]: 2024. Available from: https://extra.skr.se/skr/tjanster/rapporterochskrifter/publikationer/valfardenskompetensforsorjning20232033.85311.html

[33] Socialdepartementet [Ministry of Social Affairs]. God och nära vård: En primärvårdsreform [Good and Close Care: A Primary Care Reform]. Stockholm. 2018. Available from: https://www.regeringen.se/contentassets/85abf6c8cfdb401ea6fbd3d17a18c98e/god-och-nara-vard--en-primarvardsreform_sou-2018_39.pdf

[34] Fitzgerald DC. Aging, experienced nurses: their value and needs. Contemp Nurse. 2007;24(2):237–42. 10.5172/conu.2007.24.2.237. 17563332 10.5172/conu.2007.24.2.237

[35] Jonsson R, Lindegård A, Björk L, Nilsson K. Organizational hindrances to the retention of older healthcare workers. Nordic J Working Life Stud. 2020;10(1). 10.18291/njwls.v10i1.118679.

[36] Merkel S, Ruokolainen M, Holman D. Challenges and practices in promoting (ageing) employees working career in the health care sector - case studies from Germany, Finland and the UK. BMC Health Serv Res. 2019;19(1). 10.1186/s12913-019-4655-3. 10.1186/s12913-019-4655-3 PMC6884779 31783852

[37] The Swedish Social Insurance Agency. Sjukfrånvaro i psykiatriska diagnoser [Sickness Absence Due to Psychiatric Diagnoses]. Stockholm. 2020. Available from: https://www.forsakringskassan.se/download/18.7fc616c01814e179a9f329/1656660446139/sjukfranvaro-i-psykiatriska-diagnoser-socialforsakringsrapport-2020-8.pdf

[38] Horowitz PK, Shemesh AA, Horev T. Is there a Doctor in the house? Availability of Israeli physicians to the workforce. Isr J Health Policy Res. 2017;6(31). 10.1186/s13584-017-0157-0. 10.1186/s13584-017-0157-0 PMC5448147 28560029

[39] Orkin FK, McGinnis SL, Forte GJ, Peterson MD, Schubert A, Katz JD, et al. United States anesthesiologists over 50: retirement decision making and workforce implications. Anesthesiology. 2012;117(5):953–63. 10.1097/ALN.0b013e3182700c72. 23095532 10.1097/ALN.0b013e3182700c72

[40] Silver MP, Hamilton AD, Biswas A, Warrick NI. A systematic review of physician retirement planning. Hum Resour Health. 2016;14(1). 10.1186/s12960-016-0166-z. 10.1186/s12960-016-0166-z PMC5109800 27846852

[41] Sousa-Ribeiro M, Knudsen K, Persson L, Lindfors P, Sverke M. Meaning of working for older nurses and nursing assistants in sweden: A qualitative study. J Aging Stud. 2024;69:101230. 10.1016/j.jaging.2024.101230. 38834253 10.1016/j.jaging.2024.101230

[42] Söderbacka T, Nyholm L, Fagerström L. What is giving vitality to continue at work? A qualitative study of older health professionals’ vitality sources. Scand J Caring Sci. 2022;36(3):699–705. 10.1111/scs.13031. 34491585 10.1111/scs.13031

[43] Uthaman T, Chua TL, Ang SY. Older nurses: a literature review on challenges, factors in early retirement and workforce retention. Proceed Singapore Healthcare. 2016;25(1):50–5. 10.1177/2010105815610138

[44] Hansen V, Pit S, Honeyman P, Barclay L. Prolonging a sustainable working life among older rural GPs: solutions from the horse’s mouth. Rural Remote Health. 2013;13(2). 10.22605/RRH2369. 23781854

[45] Denton J, Evans D, Xu Q. Older nurses and midwives in the workplace: A scoping review. Collegian. 2021;28(2):222–9. 10.1016/j.colegn.2020.06.004.

[46] Stimpfel AW, Arabadjian M, Liang E, Sheikhzadeh A, Weiner SS, Dickson VV. Organization of work factors associated with work ability among aging nurses. West J Nurs Res. 2019;42(6):397–404. 10.1177/0193945919866218. 31322064 10.1177/0193945919866218 PMC6980255

[47] Storey C, Cheater F, Ford J, Leese B. Retaining older nurses in primary care and the community. J Adv Nurs. 2009;65(7):1400–11. 10.1111/j.1365-2648.2009.05009.x. 19457002 10.1111/j.1365-2648.2009.05009.x

[48] Kurashvili M, Reinhold K, Järvis M. Managing an ageing healthcare workforce: a systematic literature review. J Health Organ Manag. 2022;23(1):116–32. 10.1108/JHOM-11-2021-0411. 10.1108/JHOM-11-2021-0411 36205415

[49] Statistics Sweden. SNI 2007: Swedish Standard Industrial Classification 2007. Stocholm: SCB; 2007. Available from: https://www.scb.se/contentassets/d43b798da37140999abf883e206d0545/mis-2007-2.pdf

[50] Ludvigsson JF, Svedberg P, Olén O, Bruze G, Neovius M. The longitudinal integrated database for health insurance and labour market studies (LISA) and its use in medical research. Eur J Epidemiol. 2019;34(4):423–37. 10.1007/s10654-019-00511-8. 30929112 10.1007/s10654-019-00511-8 PMC6451717

[51] Österlund N. MiDAS - sjukpenning och rehabiliteringspenning (The MiDAS register. Sickness absence benefits)(In Swedish). The Swedish Social Insurance Agency; 2011. Available from: https://www.forsakringskassan.se/download/18.5b8b0bec183b9d817dc11f/1666183936381/dokumentation-av-midas-sjukpenning-och-rehabiliteringspenning.pdf

[52] National Board of Health and Welfare. National Cause of Death Register 2024 [cited 2025 March 27]. Available from: https://www.socialstyrelsen.se/en/statistics-and-data/registers/national-cause-of-death--register/

[53] Sveriges Kommuner och Regioner [Sweden’s municipalities and regions]. VI2000; Verksamhetsindelning för regioner 2024 [Regional Service Classification System, 2024]: 2024 [cited 2025 28 March]. Available from: https://extra.skr.se/skr/tjanster/rapporterochskrifter/publikationer/verksamhetsindelningvi2000.85551.html

[54] European Commission. Your social security rights in Sweden. Luxembourg: Directorate-General for Employment, Social Affairs and Inclusion: 2023. Available from: https://ec.europa.eu/social/BlobServlet?docId=13776&langId=en

[55] The Swedish Social Insurance Agency. Social Insurance in Figures. 2023. Stockholm: 2023. Available from: https://www.forsakringskassan.se/download/18.73da25b81888fb1e89b97d/1695274193538/social-insurance-in-figures-2023.pdf

[56] The Swedish Government Offices. Anpassning av åldersgränsen för rätten att kvarstå i anställning till riktåldern för pension [Adjustment of the statutory employment retention age to match the target pension age]: 2024. Available from: https://www.regeringen.se/contentassets/f4a857760c1e4421a0313c4b859a5ca0/kommittedirektiv-anpassning-av-aldersgransen-for-ratten-att-kvarsta-i-anstallning-till-riktaldern-for-pension.pdf

[57] Nordic Co-operation. Retirement pension in Sweden n.d. [cited 2025 27 Apr]. Available from: https://www.norden.org/en/info-norden/retirement-pension-sweden

[58] European Union. The Swedish pension system and pension projections until 2070. 2020. Available from: https://economy-finance.ec.europa.eu/system/files/2021-05/se_-_ar_2021_final_pension_fiche.pdf

[59] König S, Nerman M. Höjda åldersgränser i pensionssystemet [Raised age limits in the pension system]. Gothenbourg: Inspektionen för socialförsäkringen [Swedish Social Insurance Inspectorate]: 2023. Available from: https://isf.se/publikationer/rapporter/2023/2023-06-28-hojda-aldersgranser-i-pensionssystemet

[60] The Swedish Pensions Agency. The Swedish pension system [cited 2025 27 Apr]. Available from: https://www.pensionsmyndigheten.se/other-languages/english-engelska/english-engelska/pension-system-in-sweden

[61] Liao TF, Bolano D, Brzinsky-Fay C, Cornwell B, Fasang AE, Helske S, et al. Sequence analysis: its past, present, and future. Soc Sci Res. 2022;107:102772. 10.1016/j.ssresearch.2022.102772. 36058612 10.1016/j.ssresearch.2022.102772

[62] Studer M, Ritschard G. What matters in differences between life trajectories: A comparative review of sequence dissimilarity measures. J Royal Stat Soc Ser A: Stat Soc. 2016;179(2):481–511. 10.1111/rssa.12125.

[63] Sheppard P, Van Winkle Z. Using sequence analysis to test if human life histories are coherent strategies. Evol Hum Sci. 2020;2(e39). 10.1017/ehs.2020.38. 10.1017/ehs.2020.38 PMC10427452 37588360

[64] Velmurugan T, Santhanam T. Computational complexity between K-Means and K-Medoids clustering algorithms for normal and uniform distributions of data points. J Comput Sci. 2010;6(3). 10.3844/jcssp.2010.363.368.

[65] Gabadinho A, Ritschard G, Müller NS, Studer M. Analyzing and visualizing state sequences in R with traminer. J Stat Softw. 2011;40(4):1–37. 10.18637/jss.v040.i04.

[66] The Swedish National Mediation Office. Gender Pay Gap. 2023: What does the official wage statistics say? 2025. Available from: https://www.mi.se/app/uploads/Gender-pay-gap-2023.pdf

[67] Chen C, Shannon K, Napier S, Neville S, Montayre J. Ageism directed at older nurses in their workplace: A systematic review. J Clin Nurs. 2024;33(7):2388–411. 10.1111/jocn.17088. 38433366 10.1111/jocn.17088

[68] Gynning BE, Karlsson E, Teoh K, Gustavsson P, Christiansen F, Brulin E. Contextualising the job demands-resources model: a cross-sectional study of the psychosocial work environment across different healthcare professions. Hum Resour Health. 2024;22(1):77. 10.1186/s12960-024-00958-1. 39563348 10.1186/s12960-024-00958-1 PMC11577852

[69] Sundstrup E, Hansen ÅM, Mortensen EL, Poulsen OM, Clausen T, Rugulies R, et al. Retrospectively assessed physical work environment during working life and risk of sickness absence and labour market exit among older workers. Occup Environ Med. 2018;75(2):114–23. 10.1136/oemed-2016-104279. 28819019 10.1136/oemed-2016-104279 PMC5800344

[70] d’Errico A, Burr H, Pattloch D, Kersten N, Rose U. Working conditions as risk factors for early exit from work-in a cohort of 2351 employees in Germany. Int Arch Occup Environ Health. 2021;94(1):117–38. 10.1007/s00420-020-01566-x. 32929527 10.1007/s00420-020-01566-x PMC7826313

[71] Schram JL, Solovieva S, Leinonen T, Viikari-Juntura E, Burdorf A, Robroek SJ. The influence of occupational class and physical workload on working life expectancy among older employees. Scand J Work Environ Health. 2021;47(1):5–14. 10.5271/sjweh.3919. 32869106 10.5271/sjweh.3919 PMC7801139

[72] Farrants K, Head J, Framke E, Rugulies R, Alexanderson K. Associations between combinations of job demands and job control among 616,818 people aged 55–64 in paid work with their labour market status 11 years later: a prospective cohort study. Int Arch Occup Environ Health. 2022;95(1):169–85. 10.1007/s00420-021-01717-8. 34097108 10.1007/s00420-021-01717-8 PMC8755665

[73] Brydsten A, Stattin M. Redefining retirement: a sequence analysis of how older adults extend working life in Sweden. Ageing Soc. 2025;1-23. 10.1017/s0144686x2510024x.

[74] Brydsten A, Hasselgren C, Stattin M, Larsson D. The road to retirement: A life course perspective on labor market trajectories and retirement behaviors. Work Aging Retire. 2025;11(1):1–12. 10.1093/workar/waad024.

[75] Statistics Sweden. Det statistiska registrets framställning och kvalitet: Longitudinell integrationsdatabas för sjukförsäkrings- och arbetsmarknadsstudier (LISA) [Production and Quality of the Statistical Register: Longitudinal Integration Database for Health Insurance and Labour Market Studies (LISA)]. 2021. Available from: https://www.scb.se/contentassets/0521204f13e649299dec73f091e691e0/lisa-am9901_dokstar_2021.pdf

[76] Brooke HL, Talback M, Hornblad J, Johansson LA, Ludvigsson JF, Druid H, et al. The Swedish cause of death register. Eur J Epidemiol. 2017;32(9):765–73. 10.1007/s10654-017-0316-1. 28983736 10.1007/s10654-017-0316-1 PMC5662659

[77] Ludvigsson JF, Håberg SE, Knudsen GP, Lafolie P, Zoega H, Sarkkola C, et al. Ethical aspects of registry-based research in the nordic countries. Clin Epidemiol. 2015;23(7):491–508. 10.2147/CLEP.S90589. 10.2147/CLEP.S90589 PMC4664438 26648756

