# Supplementary material for: Older health and social care workers’ labour market patterns: a 16-year longitudinal study from ages 61–65 to 76–80
Source: BMC Health Serv Res. 2025 Nov 26;25:1537. doi: 10.1186/s12913-025-13707-4 (PMC12659274; doi:10.1186/s12913-025-13707-4)
Supplement: Supplementary file 1 — Supplementary Material 1 [file 12913_2025_13707_MOESM1_ESM.pdf]

Supplementary material to

Older Health and Social Care Workers' Labour Market Patterns: A 16-Year Longitudinal Study from Ages 61-65 to 76-80

Authors: Aleksiina Martikainen[1]*, Kristina Alexanderson[1], Pia Svedberg[1] & Kristin Farrants[1]

[1] Division of Insurance Medicine, Department of Clinical Neuroscience, Karolinska Institutet, SE-17177 Stockholm, Sweden

* Corresponding author: aleksiina.martikainen@ki.se

Supplementary Section A

Methodological Aspects

Supplementary Figure 1

Flowchart illustrating inclusion criteria

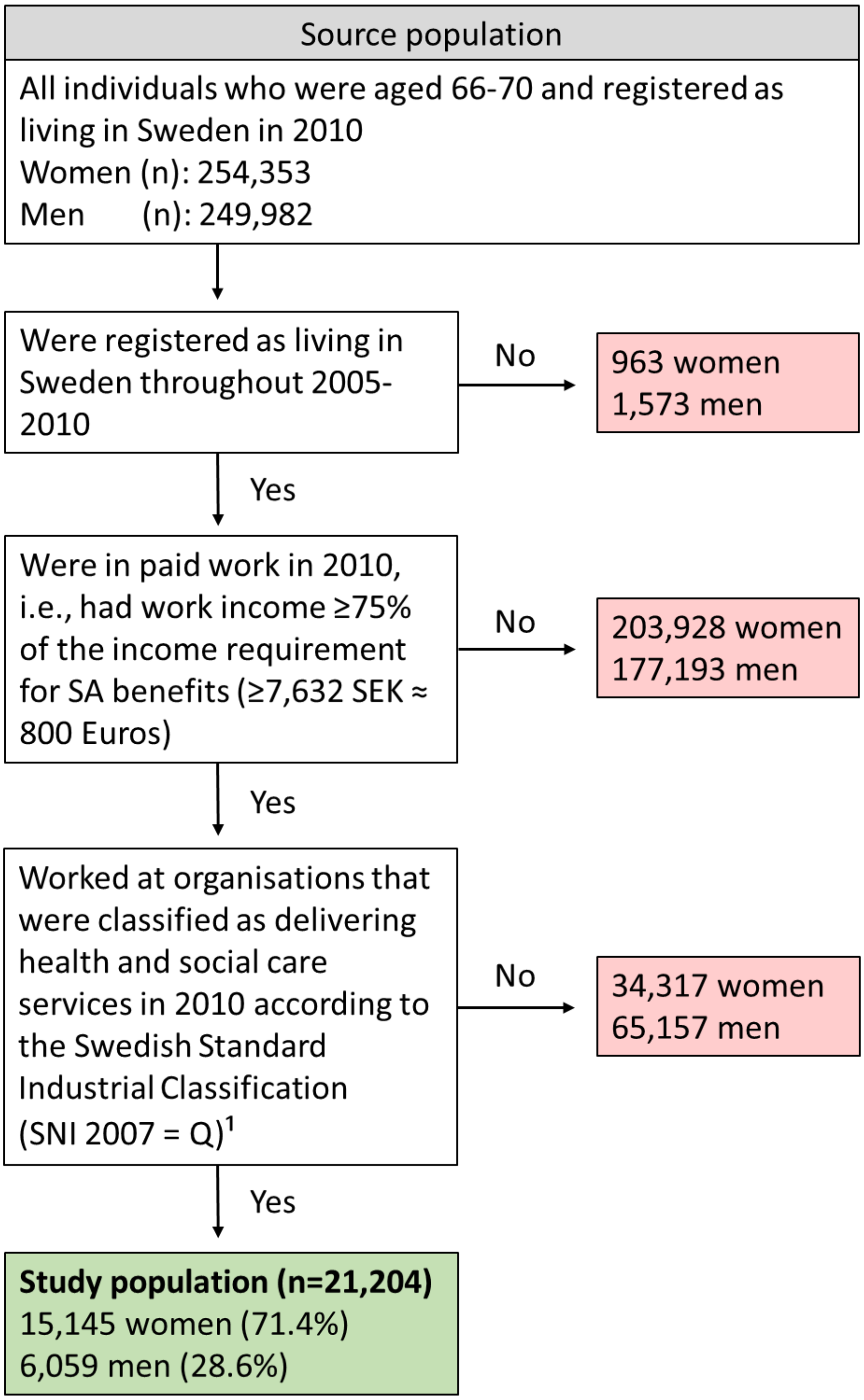

Note. [1] Statistics Sweden. *Swedish Standard Industrial Classification (SNI).* www.scb.se/en/documentation/classifications-and-standards/swedish-standard-industrial-classification-sni/

Supplementary Table 1

Validity scores for different number of clusters using OMspell as a dissimilarity measure

**Women**										
Number of clusters	PBC	HG	HGSD	ASW	ASWw	CH	R2	CHsq	R2sq	HC
2	0.43	0.63	0.63	0.52	0.52	7029.74	0.32	10238.07	0.40	0.21
3	0.66	0.89	0.89	0.63	0.63	9573.35	0.56	28830.07	0.79	0.04
4	0.68	0.95	0.95	0.65	0.65	8150.49	0.62	28712.58	0.85	0.02
5	0.58	0.91	0.90	0.58	0.58	7624.57	0.67	26178.61	0.87	0.04
6	0.59	0.92	0.91	0.58	0.58	6977.31	0.70	27176.36	0.90	0.03
7	0.53	0.92	0.91	0.48	0.48	6473.97	0.72	25585.11	0.91	0.03
8	0.52	0.92	0.92	0.50	0.50	6033.40	0.74	23983.12	0.92	0.03
9	0.46	0.89	0.89	0.50	0.50	5880.30	0.76	22181.92	0.92	0.04
**Men**										
Number of clusters	PBC	HG	HGSD	ASW	ASWw	CH	R2	CHsq	R2sq	HC
2	0.70	0.84	0.84	0.62	0.62	3777.86	0.38	8305.50	0.58	0.06
3	0.75	0.92	0.92	0.63	0.63	3296.19	0.52	7532.09	0.71	0.04
4	0.78	0.97	0.97	0.68	0.68	3017.80	0.60	8901.47	0.82	0.01
5	0.61	0.87	0.87	0.53	0.53	2783.06	0.65	7891.44	0.84	0.06
6	0.64	0.92	0.92	0.55	0.55	2715.48	0.69	10281.32	0.89	0.03
7	0.52	0.85	0.85	0.50	0.50	2533.34	0.72	9140.96	0.90	0.05
8	0.50	0.86	0.86	0.54	0.54	2413.41	0.74	8803.35	0.91	0.05
9	0.47	0.89	0.88	0.45	0.45	2246.37	0.75	8305.51	0.92	0.04

Note. PBC = Point-Biserial Correlation. HG = Hubert's Gamma. HGSD= Hubert's Gamma (Somers' D). ASW = Average Silhouette Width. ASWw = Average Silhouette Width (weighted). CH = Calinski-Harabasz Index. R2 = Pseudo-R-squared. CHsq = Calinski-Harabasz Index Squared. R2sq = Pseudo-R-squared Squared. HC = Hubert's C Index. The chosen cluster solution is highlighted in grey. The OMspell was conducted with zero expansion cost, and the cost of insertion/deletion was set to *max (substitution cost/2)*.

Supplementary Section B

Labour Market States, Transition Probabilities, Entropy, and Mean Time in States among All Women and Men

Supplementary Table 2

Numbers and percentages of women and men in the five different labour market states per year.

State →	Women (n=15,145)										Men (n=6,059)									
	Medium/high work income		*Low work income*		*SA/DP*		*No work income*		*Emigrated or dead*		*Medium/high work income*		*Low work income*		*SA/DP*		*No work income*		*Emigrated or dead*	
Year ↓	n	%	n	%	n	%	n	%	n	%	n	%	n	%	n	%	n	%	n	%
2005	9,885	65.3	637	4.2	3,973	26.2	650	4.3	0	0	4,074	67.2	502	8.3	985	16.3	498	8.2	0	0
2006	9,589	63.3	1,124	7.4	3,680	24.3	752	5	0	0	3,928	64.8	734	12.1	889	14.7	508	8.4	0	0
2007	9,419	62.2	1,883	12.4	3,072	20.3	771	5.1	0	0	3,782	62.4	1,038	17.1	748	12.3	491	8.1	0	0
2008	8,981	59.3	2,977	19.7	2,366	15.6	821	5.4	0	0	3,614	59.6	1,414	23.3	568	9.4	463	7.6	0	0
2009	8,167	53.9	4,812	31.8	1,422	9.4	744	4.9	0	0	3,432	56.6	1,957	32.3	331	5.5	339	5.6	0	0
2010	6,766	44.7	7,908	52.2	471	3.1	0	0	0	0	3,129	51.6	2,812	46.4	118	1.9	0	0	0	0
2011	4,982	32.9	6,070	40.1	198	1.3	3,834	25.3	61	0.4	2,608	43	2,300	38	70	1.2	1,031	17	50	0.8
2012	3,858	25.5	5,135	33.9	85	0.6	5,936	39.2	131	0.9	2,215	36.6	2,063	34	39	0.6	1,627	26.9	115	1.9
2013	3,173	21	4,455	29.4	78	0.5	7,217	47.7	222	1.5	1,970	32.5	1,930	31.9	46	0.8	1,935	31.9	178	2.9
2014	2,742	18.1	3,913	25.8	73	0.5	8,078	53.3	339	2.2	1,739	28.7	1,855	30.6	36	0.6	2,173	35.9	256	4.2
2015	2,346	15.5	323	21.4	57	0.4	9,041	59.7	465	3.1	1,463	24.1	163	27	35	0.6	2,583	42.6	343	5.7
2016	2,023	13.4	2,859	18.9	39	0.3	9,610	63.5	614	4.1	1,298	21.4	1,476	24.4	15	0.2	2,838	46.8	432	7.1
2017	1,691	11.2	2,544	16.8	36	0.2	10,103	66.7	771	5.1	1,109	18.3	1,385	22.9	24	0.4	3,015	49.8	526	8.7
2018	1,393	9.2	2,319	15.3	18	0.1	10,458	69.1	957	6.3	893	14.7	1,328	21.9	20	0.3	3,164	52.2	654	10.8
2019	1,106	7.3	1,964	13	17	0.1	10,922	72.1	1,136	7.5	709	11.7	1,162	19.2	10	0.2	3,385	55.9	793	13.1
2020	628	4.1	1,650	10.9	17	0.1	11,472	75.7	1,378	9.1	514	8.5	938	15.5	13	0.2	3,678	60.7	916	15.1

Note. SA/DP = sickness absence and/or disability pension. These percentages are visually illustrated in Figure 1 in the manuscript. Among individuals in the *Emigrated or dead* state, 52 women and 22 men emigrated in total; the others died.

Supplementary Table 3

Transition probabilities from one labour market state (row) to another state (column) by sex

**Women**					
	➡ Medium/high work income	➡ Low work income	➡ SA/DP	➡ No work income	➡ Emigrated or dead
Medium/high work income ➡	75.6%	15.8%	4.6%	3.8%	0.2%
Low work income ➡	7.3%	67.7%	0.2%	24.4%	0.4%
SA/DP ➡	32.7%	12.9%	50.2%	4%	0.1%
No work income ➡	0.5%	4.7%	0.3%	93.2%	1.3%
Emigrated or dead ➡	0%	0%	0%	0.1%	99.9%
**Men**					
	➡ Medium/high work income	➡ Low work income	➡ SA/DP	➡ No work income	➡ Emigrated or dead
Medium/high work income ➡	80.8%	11.9%	2.6%	4.2%	0.4%
Low work income ➡	7.1%	73.3%	0.2%	18.3%	1.1%
SA/DP ➡	33.3%	14%	47%	5.4%	0.4%
No work income ➡	1.4%	8%	0.5%	88%	2.1%
Emigrated or dead ➡	0%	0%	0%	0.1%	99.9%

Note. SA/DP = sickness absence and/or disability pension. Probabilities are expressed in percentages.

Supplementary Figure 2

Entropy and yearly distribution of labour market states among all women (left) and men (right)

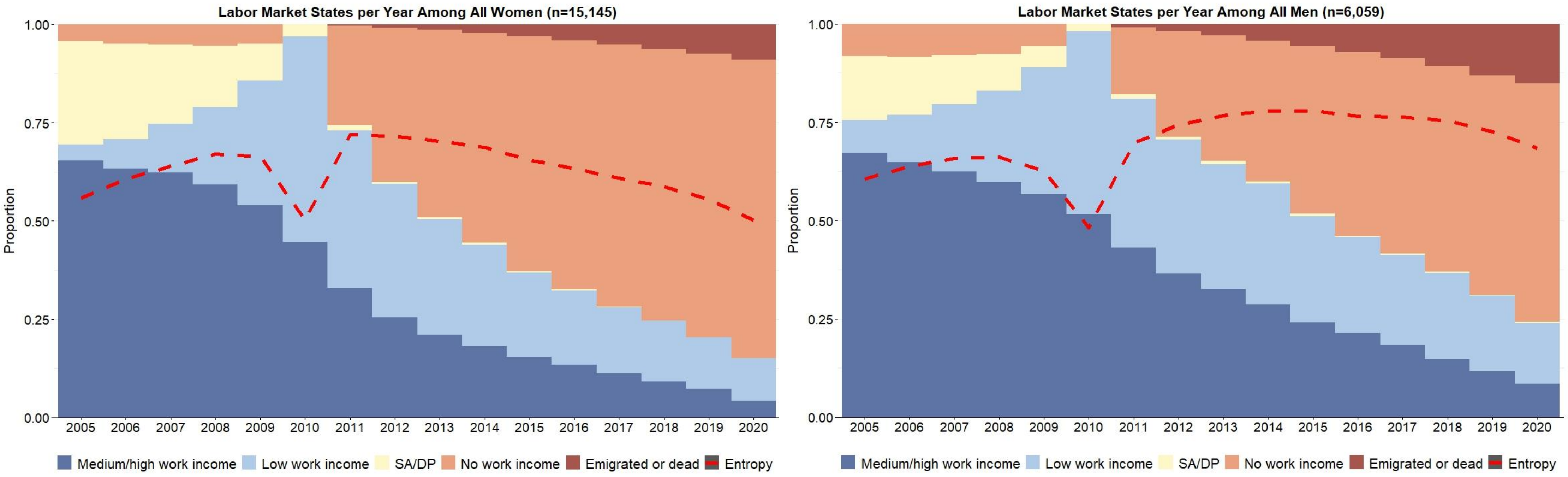

Note. SA/DP = sickness absence and/or disability pension.

Supplementary Figure 3
Mean time spent in each state among all women (left) and men (right)

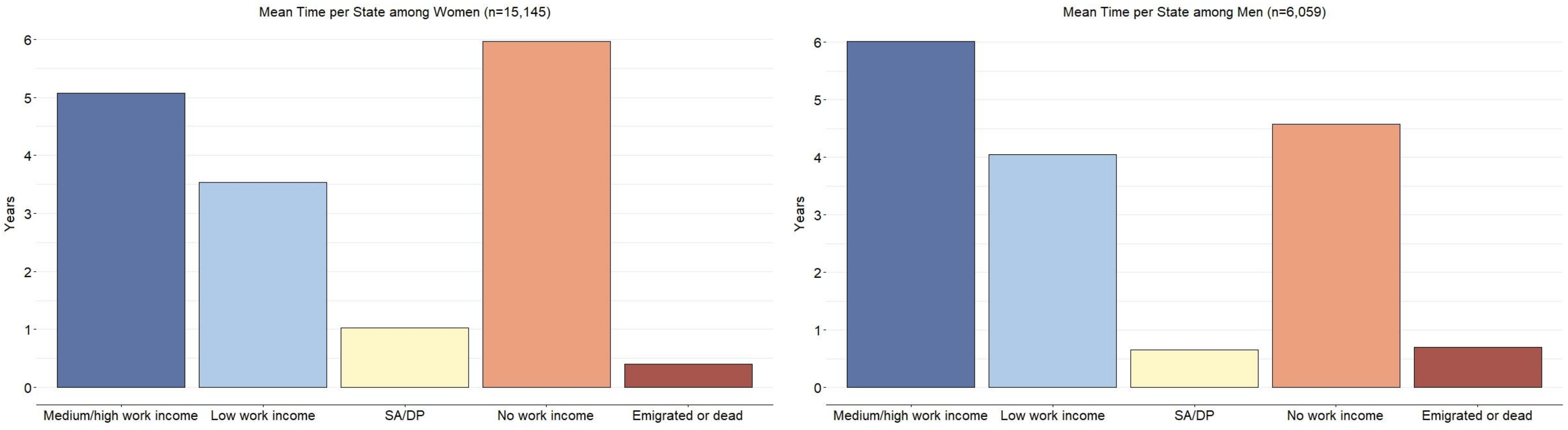

Note. SA/DP = sickness absence and/or disability pension.

Supplementary Section C

Labour Market Sequences among All Women and Men

Supplementary Figure 4. Individual labour market sequences among all women (n=15,145; left panels) and all men (n=6,059; right panels)

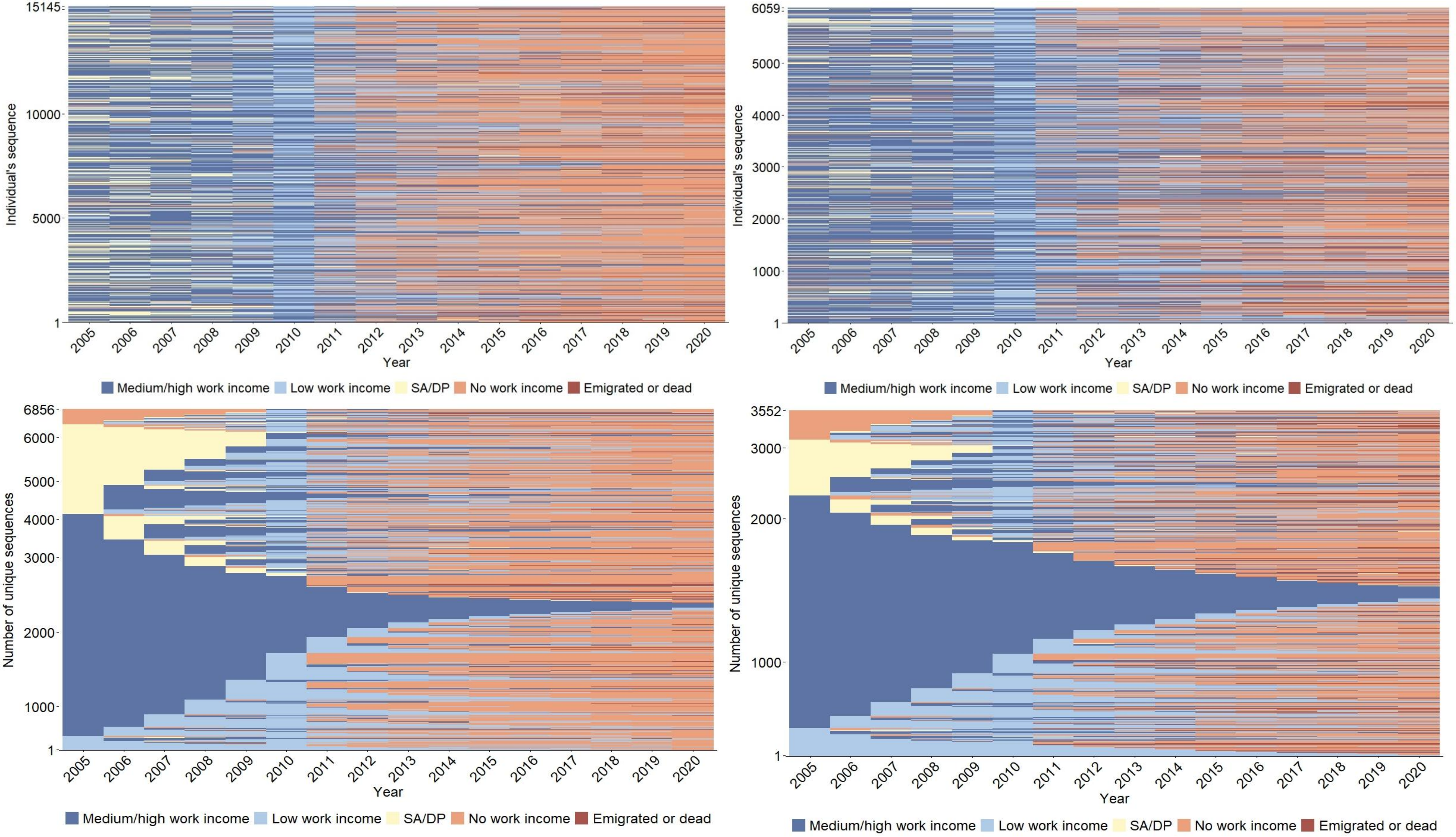

Note. SA/DP = sickness absence and/or disability pension. The upper panels show individual sequences (one line per person). In the lower panels, individuals who share an identical sequence are aggregated into one horizontal line, resulting in 6,856 unique sequences among women and 3,552 unique sequences among men (sorted by the first year). Women are shown in the left panels and men in the right panels.

Supplementary Figure 5

Ten most common sequences among all women (left) and men (right)

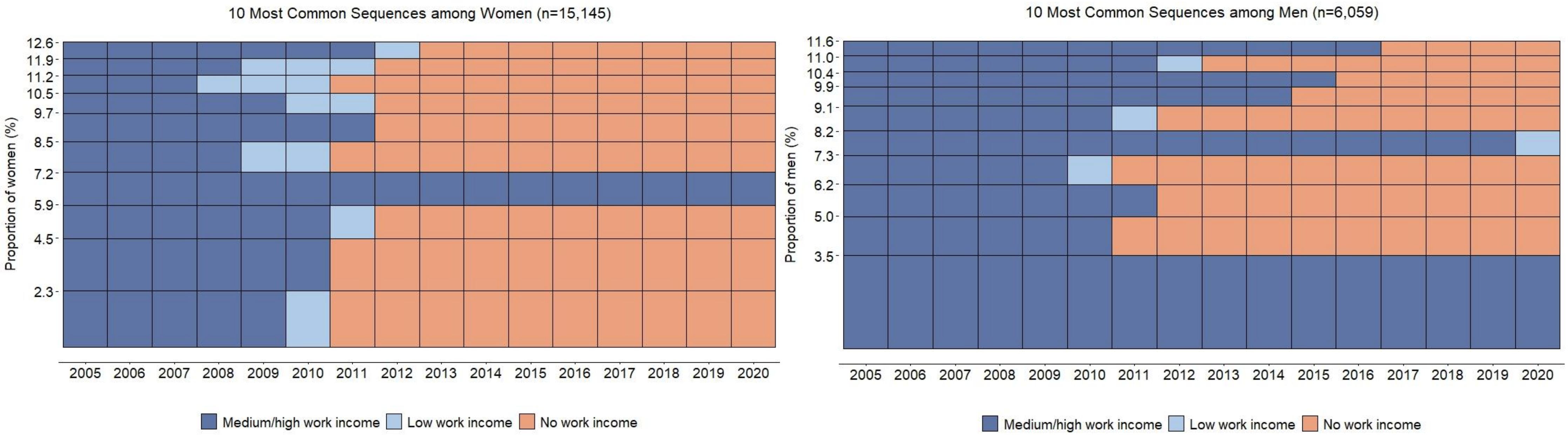

Supplementary Section D

Cluster-level Sequence Details

Supplementary Figure 6a

Ten most common sequences per cluster among women

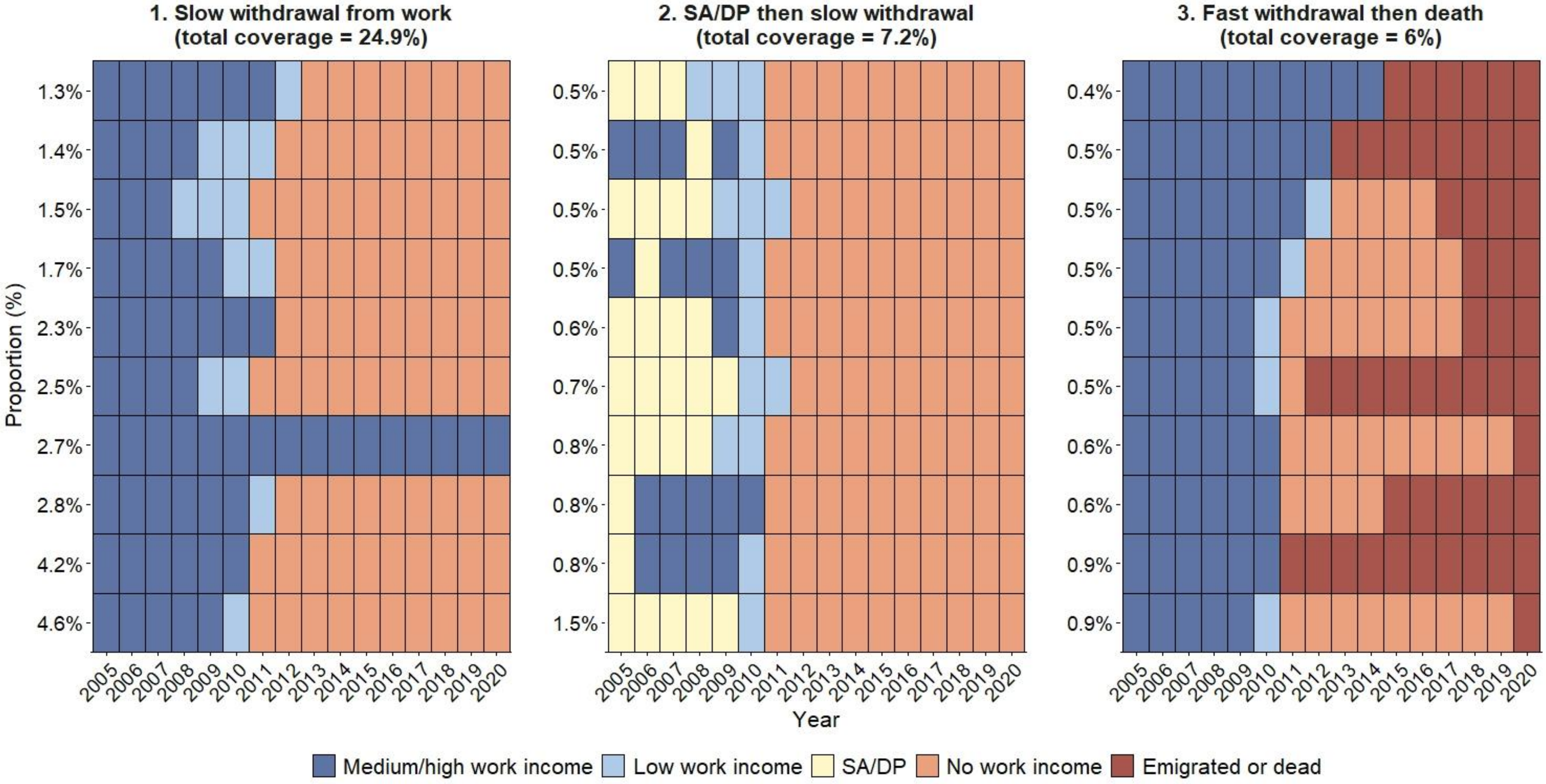

Note. SA/DP = sickness absence and/or disability pension.

Supplementary Figure 6b

Ten most common sequences per cluster among men

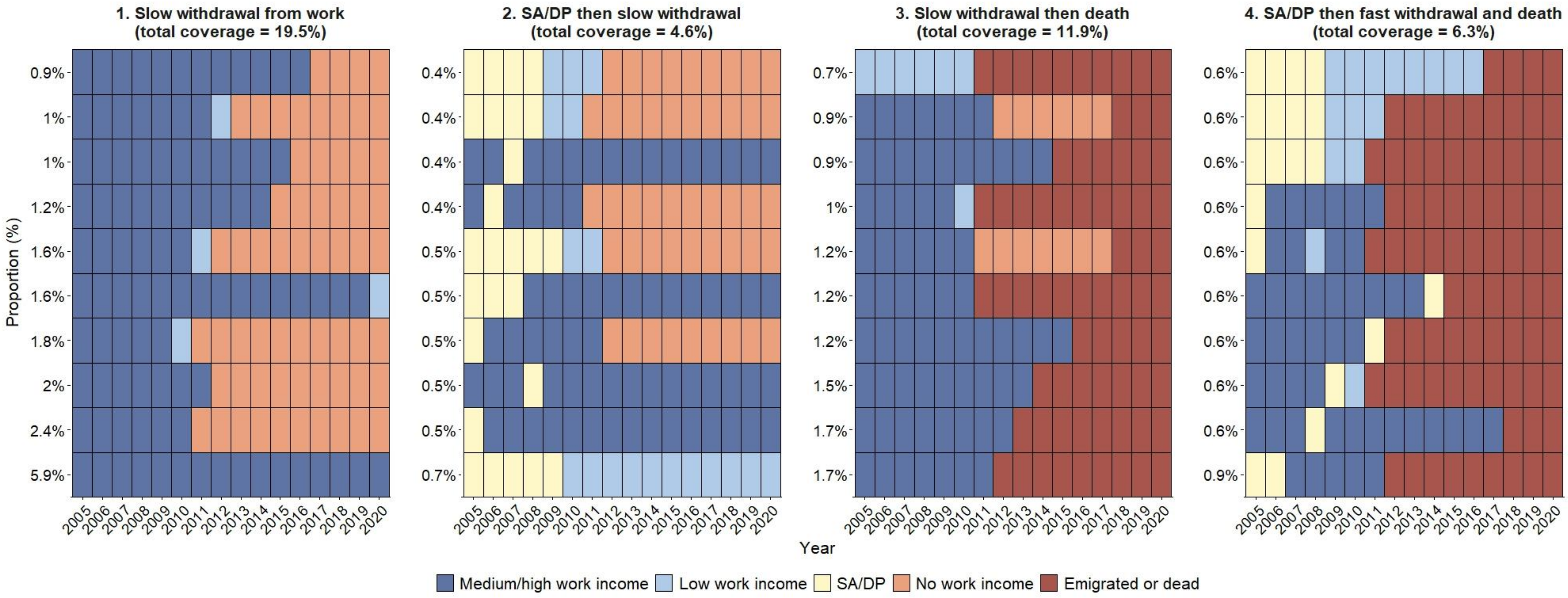

Note. SA/DP = sickness absence and/or disability pension.

Supplementary Section E

Alternative Cluster Solution

Supplementary Figure 7

Alternative solution with four clusters among women

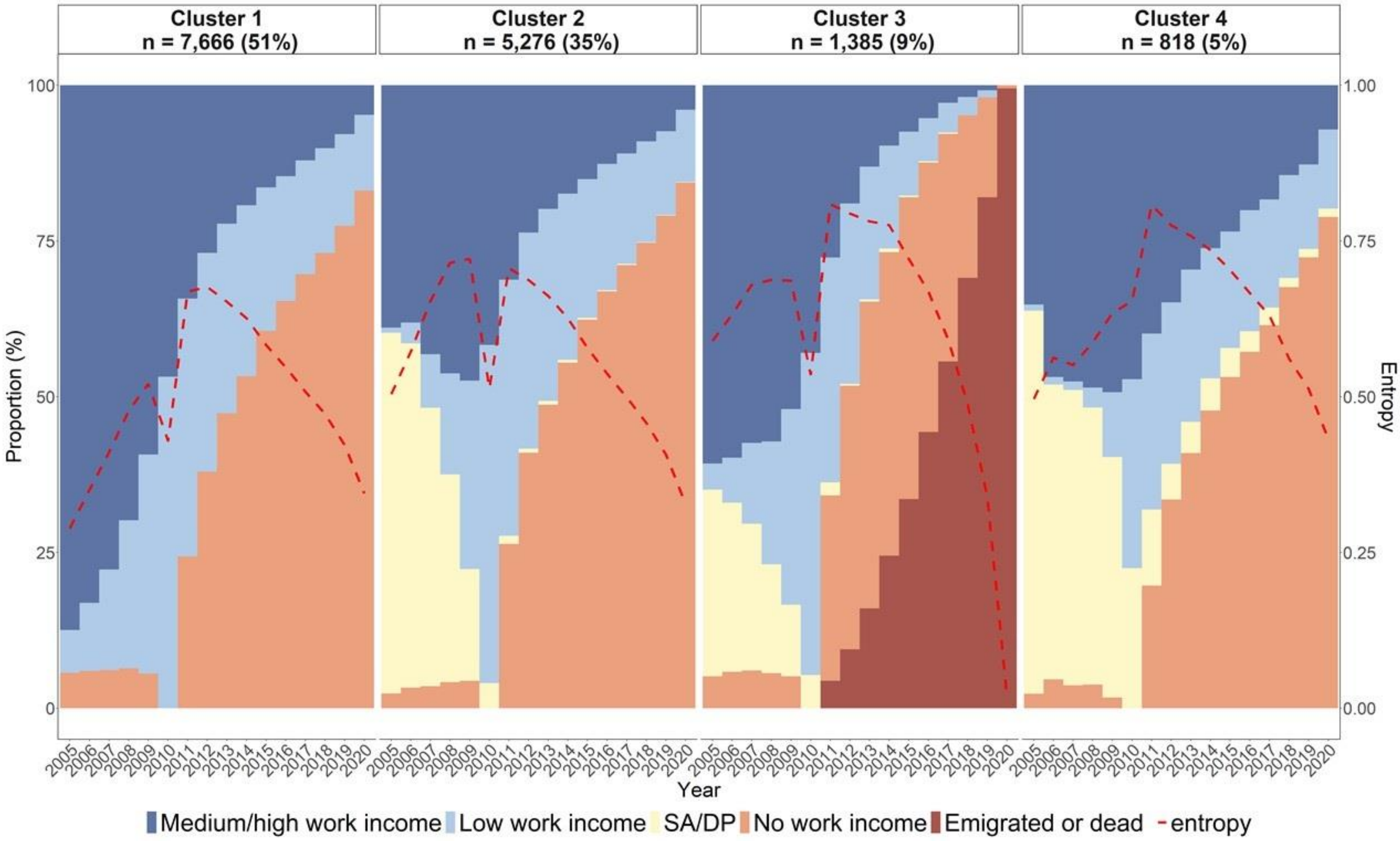

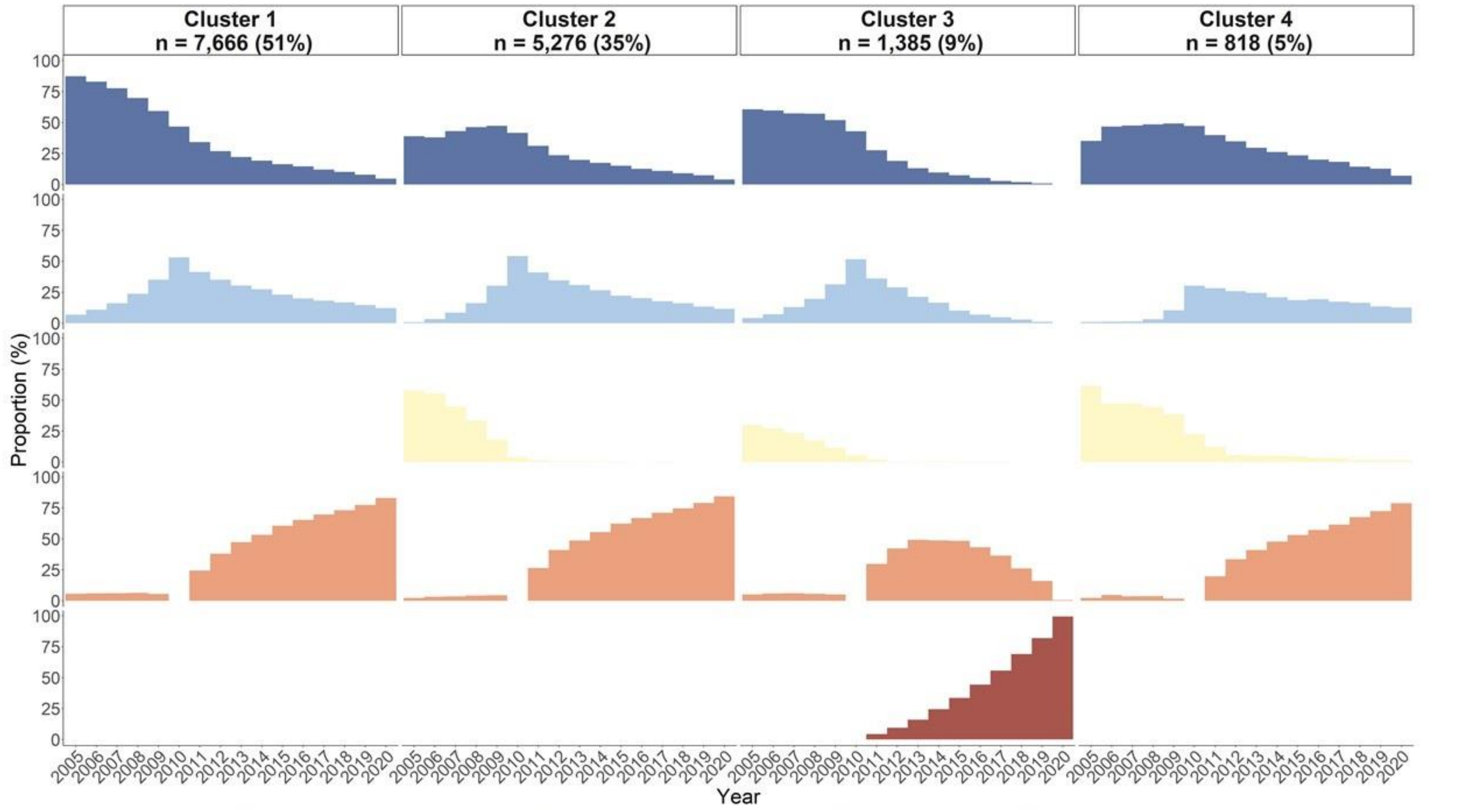

Note. SA/DP = sickness absence and/or disability pension.
